# Indolamine accumulation and *TDC*/*T5H* expression profiles reveal the complex and dynamic regulation of serotonin biosynthesis in tomato (*Solanum lycopersicum* L.)

**DOI:** 10.3389/fpls.2022.975434

**Published:** 2022-08-11

**Authors:** Mauro Commisso, Stefano Negri, Elisa Gecchele, Emanuela Fazion, Cecilia Pontoriero, Linda Avesani, Flavia Guzzo

**Affiliations:** Biotechnology Department, University of Verona, Verona, Italy

**Keywords:** tryptamine, serotonin, fruit indolamines, tryptophan decarboxylase, tryptamine 5-hydroxylase, biosynthesis pathway, *Solanum lycopersicum*

## Abstract

Tryptamine and serotonin are indolamines that fulfill diverse biological functions in all kingdoms of life. Plants convert l-tryptophan into tryptamine and then serotonin *via* consecutive decarboxylation and hydroxylation reactions catalyzed by the enzymes tryptophan decarboxylase (TDC) and tryptamine 5-hydroxylase (T5H). Tryptamine and serotonin accumulate to high levels in the edible fruits and seeds of many plant species, but their biological roles in reproductive organs remain unclear and the metabolic pathways have not been characterized in detail. We identified three *TDC* genes and a single *T5H* gene in tomato (*Solanum lycopersicum* L.) by homology-based screening and confirmed their activity by heterologous expression in *Nicotiana benthamiana*. The co-analysis of targeted metabolomics and gene expression data revealed complex spatiotemporal gene expression and metabolite accumulation patterns that suggest the involvement of the serotonin pathway in multiple biological processes. Our data support a model in which *SlTDC1* allows tryptamine to accumulate in fruits, *SlTDC2* causes serotonin to accumulate in aerial vegetative organs, and *SlTDC3* works with *SlT5H* to convert tryptamine into serotonin in the roots and fruits.

## Introduction

Indolamines are ubiquitous tryptophan-derived indole alkaloids found in all kingdoms of life, albeit with extremely diverse distribution profiles and biological functions (Roshchina, 2010). The ability of many plants to produce the indolamines tryptamine and serotonin has driven research into the corresponding metabolic pathways, in many cases due to the role of these products as intermediates in the synthesis of specialized metabolites with pharmacological activity, such as antitumor indole alkaloids and melatonin (Arnao and Hernández-Ruiz, 2018). Recent literature provides evidence that plant indolamines are involved in various physiological processes, including development and biotic/abiotic stress responses, leading some authors to consider serotonin and melatonin as a new class of phytohormones (Bhatla, 2018; Arnao and Hernández-Ruiz, 2019; Erland et al., 2019; Akula and Mukherjee, 2020; Sun et al., 2021). In contrast, the potential biological functions of tryptamine have received far less attention because it is widely considered as an intermediate rather than an end-product, even though both tryptamine and serotonin accumulate to high levels in particular organs of some plant species (Negri et al., 2021). For example, melatonin is found in the fleshy fruits and seeds of some plants with concentrations in the ng g^−1^ FW range (Nawaz et al., 2016) whereas the concentration of tryptamine and serotonin can reach hundreds of μg g^−1^ FW (Huang and Mazza, 2011; Islam et al., 2016; Yılmaz et al., 2019). This 1,000-fold difference in abundance represents a significant investment of resources to ensure the accumulation of tryptamine and serotonin, which must therefore provide some advantage during the reproductive phase. Moreover, dynamic indolamine accumulation profiles have been reported throughout fruit development and ripening, with corresponding changes in gene expression profiles (Hano et al., 2017; Commisso et al., 2019).

In plants, tryptamine is synthesized from l-tryptophan by the enzyme tryptophan decarboxylase (TDC), a cytosolic pyridoxal-5′-phosphate-dependent type II aromatic l-amino acid decarboxylase (AADC). This diverts carbon and nitrogen from primary metabolism into the indole alkaloid pathway of specialized metabolism. The first plant *TDC* gene was found in the Madagascar periwinkle *Catharanthus roseus* (De Luca et al., 1989) and several orthologs have been identified by homology-based screening and functionally characterized by expression in *Escherichia coli*. Most plants have a single *TDC* gene, including *C. roseus* (Goddijn et al., 1994), whereas two *TDC* genes are found in *Aegilops variabilis*, *Camptotheca acuminata*, *Capsicum annuum* and *Ophiorrhiza pumila* (López-Meyer and Nessler, 1997; Yamazaki et al., 2003; Park et al., 2009; Li et al., 2016; You et al., 2020). *Oryza sativa* has three *TDC* genes (Kang et al., 2007a), and tryptamine is the first committed step in the rice indolamine pathway leading to serotonin and melatonin production at different subcellular levels (Back et al., 2016).

A cytochrome P450 monooxygenase located in the membrane of the endoplasmic reticulum (ER) is the tryptamine 5′-hydroxylase (T5H) that converts tryptamine into serotonin (5-hydroxytryptamine). Only the rice *T5H* gene has been characterized thus far, thanks to the discovery of *sl* mutants that accumulate tryptamine due to a deficit in *T5H* expression (Kang et al., 2007b; Fujiwara et al., 2010). Melatonin is then synthesized in a two-step process that involves the *N*-acetylation and methylation (in either order) of serotonin (Kang et al., 2011, 2013; Byeon et al., 2015). In parallel, other tryptamine and serotonin derivatives can be synthesized in plants including their *N*-cinnamoyl amides, in which the indolamine backbone is joined to the phenylpropanoid skeleton of cinnamic acid derivatives (mainly ferulic and coumaric acids) to generate powerful antioxidants with likely defensive functions against fungal pathogens and UV stress (Zeiss et al., 2020).

Despite the existence of alternative metabolic routes that produce melatonin *via* the hydroxytryptophan intermediate might not be excluded, the presence of *TDC* and *T5H* genes is a reasonable hypothesis in all plants that produce tryptamine and serotonin, or their downstream metabolites. For example, we recently identified *TDC* genes in green kiwifruit (*Actinidia deliciosa*) and yellow kiwifruit (*Actinidia chinensis*) based on the bulk accumulation of tryptamine and serotonin in both unripe and ripe berries (Commisso et al., 2019). However, given their arboreal nature, metabolic interventions to address the biological role of plant indolamines in the reproductive organs of these species might be challenging. As an alternative, tomato (*Solanum lycopersicum*) is a model fleshy fruit crop that is known to produce both tryptamine and serotonin. A putative TDC was purified from the cultivar Big Boy nearly 50 years ago (Gibson et al., 1972) but the molecular basis of tryptamine and serotonin biosynthesis in tomato was then largely ignored until more recent interest in melatonin. Two putative TDCs and one T5H were then postulated to exist in tomato (Hano et al., 2017), leading to the prediction of five putative *TDC* genes based on the protein sequences (Pang et al., 2018), and the very recent *in vivo* characterization of the isoform SlTDC1 (Tsunoda et al., 2021).

Here we report for the first time the identity of all biosynthetic genes involved in the tomato serotonin pathway, comprising three *TDC* genes and a single *T5H* gene. The genes were functionally characterized by transient expression in *Nicotiana benthamiana*, and the expression profiles were associated with untargeted metabolomics data as a means to link gene expression to enzymatic activity and metabolite accumulation. We also investigated the spatial and temporal profiles of *TDC* and *T5H* gene expression in relation to tryptamine and serotonin production in the whole tomato plant, providing insight into the physiological functions of these indolamines.

## Materials and methods

### Homology-based identification of SlTDC and SlT5H candidates and computational analysis

The protein sequences of functionally characterized plant TDCs and T5Hs were downloaded from NCBI (Supplementary Table 1) and used as blastp queries against tomato proteins deposited in SGNdb (ITAG release 3.20)^1^ with the parameters percentage identity >50%, alignment score > 500, and the presence of the protein candidates in the output list of all queries. All alignments of nucleotide and amino acid sequences were performed by using Clustal Omega with default parameters.^2^ To build the phylogenetic tree in which we reported the putative tomato TDCs and the plant AADCs listed in Supplementary Table 1 we used a MUSCLE alignment (MEGA X) applying the neighbor-joining method and bootstrap analysis (1,000 iterations).

### Plant material and growth conditions for functional characterization experiments

Tomato plants (*Solanum lycopersicum* cv. Micro-Tom) were grown in a growth chamber at 25°C with a photoperiod 15 h: 9 h (light: dark), resulting in blooming after 1 month and a complete life cycle of about 4 months. *Nicotiana benthamiana* plants were grown in a growth chamber at 25°C with a photoperiod 13 h: 11 h (light: dark) until they reached about 30 cm in height before transient expression experiments. Both species were grown in a 4:1 mixture of peat and sand.

### RNA extraction and cDNA synthesis

Total RNA was extracted from 50 to 100 mg frozen powdered plant material (tissue dependent) using the Spectrum Plant Total RNA Kit (Sigma-Aldrich, St. Louis, United States). RNA quality was checked through NanoDrop 8,000 spectrophotometer (Thermo Fisher Scientific, Waltham, United States) and samples beyond the range Abs_260_/Abs_280_ = 1.8–2.2 and Abs_260_/Abs_230_ = 1.7–2.2 were mixed with one third of a volume of 7.5 M LiCl (Thermo Fisher Scientific) and resolubilized. RNA integrity was checked by 2% (w/v) agarose gel electrophoresis. Then, according to the manufacturers’ instructions, 2 μg total RNA was subjected to DNase treatment with Turbo DNase (Thermo Fisher Scientific) and used to prepare cDNA through the SuperScript^™^ III First-Strand Synthesis System (Thermo Fisher Scientific).

### Molecular cloning of candidate SlTDCS and SlT5H genes in vector pK7WG2

The tomato *SlTDCs* and *SlT5H* sequences were cloned by using specific primers (Supplementary Tables 2, 3) from the cDNA obtained from total RNA of leaves and flowers at anthesis sampled from 2-month-old Micro-Tom plants, and of mature-green fruits collected at 20 days post anthesis. The amplification products were directionally cloned into vector pENTR/D-TOPO for sequencing, and verified inserts were transferred to the binary overexpression vector pK7WG2 by LR recombination (Karimi et al., 2002).

### Transient expression and untargeted HPLC-ESI-MS analysis

Vectors containing the *SlTDC* and *SlT5H* sequences or the negative control marker *GFP* were introduced into *Agrobacterium tumefaciens* strain EHA105. A positive control containing the *Actinidia chinensis TDC* gene (*AcTDC*) was also available (Commisso et al., 2019) and the *Oryza sativa T5H* gene (GenBank AK071599) was prepared by GeneArt Gene Synthesis (Thermo Fisher Scientific). Transient expression was carried out as previously described (Gecchele et al., 2015). Briefly, three leaves of a 5-week-old *N. benthamiana* plant were syringe infiltrated with a bacterial suspension (OD_600_ = 0.9). Three plants were treated with each construct and one leaf (a biological replicate) was collected from each plant after 3 days, then another after 4 days, and another after 5 days. The leaves were immediately homogenized in liquid nitrogen, followed by extraction and untargeted metabolomics analysis by HPLC-ESI-MS as previously described (Commisso et al., 2019).

### LC–MS data processing and multivariate statistical analysis

Chromatograms and mass spectra were processed using Data Analysis v3.2 (Bruker, Billerica, United States) and metabolites were identified based on *m/z*, retention time and MS/MS fragmentation pattern. The chromatograms were converted to netCDf files for peak alignment and area extraction using MZmine.^3^ The resulting feature matrix was analyzed using SIMCA v13.0 (Umetrics, Umea, Sweden). Pareto scaling was applied to all analytical methods. Unsupervised principal component analysis (PCA) was used to identify the major clusters defined by the samples before supervised orthogonal partial least squares discriminant analysis (OPLS-DA/O2PLS-DA). OPLS-DA/O2PLS-DA models were cross-validated by ANOVA with a threshold of *p* < 0.01.

### Plant material for targeted metabolomics and gene expression analysis

Tomato plants at the flowering stage grown in the above-mentioned conditions were used for sampling roots, hypocotyl, cotyledons, first leaves following cotyledons, true leaves from six different nodes, the stem (separate internodes), flower buds and flowers at anthesis. Roots were further separated into the primary root, primary-proximal secondary root and primary-distal secondary root. Another group of plants was grown to complete the life cycle and, following fruit set, fruits were collected at various developmental and ripening phases based on phenological observation: immature-green (berry still growing), mature-green (fruit at maximum size), early breaker (green-white/yellow fruit), late breaker (red-orange turning), ripe and over-ripe. Immature-green and mature-green fruits were pooled for the analysis of exocarp (peel), mesocarp (flesh) and seeds, and the same strategy was applied to ripe and over-ripe fruits. All samples consisted of three biological replicates, each represented by a pool of material collected from five different plants. The samples were immediately frozen in liquid nitrogen and ground to a fine powder using an A11 basic analytical mill (IKA-Werke, Staufen, Germany). The powder was stored at −80°C.

### Quantification of tryptamine and serotonin

We extracted 100 mg frozen powder representing each sample in 1 ml 2:1 (v/v) LCMS-grade chloroform-methanol (Honeywell, Charlotte, United States) then added 150 μl LCMS-grade ultrapure water (Honeywell) to allow phase separation. The samples were mixed vigorously for 30 s, sonicated at 40 kHz in a Sonica Ultrasonic Cleaner (Soltec, Milan, Italy) at 4°C for 15 min, and centrifuged (16,000 × g, 15 min, 4°C). The upper methanolic phase was recovered and the volume of each sample was recorded allowing appropriate dilution (according to the original tissue) in methanol and subsequent 1:2 dilution with an aqueous solution containing known amounts of the standards d_4_-tryptamine and d_4_-serotonin (Sigma-Aldrich). The final methanol-aqueous mixtures were passed through a Minisart RC4 filter (0.2 μm pores) and, for each extract, 1 μl was injected into an Acquity I Class UPLC system (Waters, Milford, United States) equipped with an Acquity UPLC BEH C18 column (Waters) kept at 30°C. The separation of the metabolites in the extracts was performed *via* reverse phase chromatography through establishment of a 20-min polarity gradient among two mobile phases (A: 0.1% formic acid in water, B: acetonitrile), which flowed at 0.350 ml min^−1^. The initial conditions were 99% A and 1% B, and the following elution profile was applied: 0–1 min, 1% B; 1 min – 10 min, 1–40% B; 10 min – 13 min 30 s, 40–70% B; 13 min 30 s – 14 min, 70–99% B; 14 min – 16 min, 99% B; 16 min – 16 min 6 s, 99–1% B (initial conditions). The system was equilibrated in 99% A and the elution was complete after 20 min.

The UPLC was connected online to a Xevo G2-XS QToF mass spectrometer (Waters) featuring an electrospray ionization (ESI) source operating in positive ionization mode within the range 50–2000 *m/z*. The ion source parameters were: capillary = 0.8 kV, sampling cone = 40 V, source offset = 80 V, source temperature = 120°C, desolvation temperature = 500°C, cone gas flow rate = 50 l h^−1^ and desolvation gas flow rate = 1,000 l h^−1^. Nitrogen gas was used for the nebulizer and desolvation, whereas argon was used to induce collision-induced dissociation. An MS method was created to acquire data in continuum mode using a fixed collision energy in two scan functions. In function 1, the low energy was disabled, whereas in function 2, the high energy was set to 35 V. In both functions, the Xevo G2-XS was set to perform the analysis with a scan time of 0.3 s. The lock mass solution used as “calibrator” to verify MS accuracy consisted of a 100 pg. μL^−1^ leucine-enkephalin solution (Waters) injected at a flow rate of 10 μl min^−1^, generating a signal of 556.2771 *m/z* in positive mode and 554.2615 *m/z* in negative mode. A quality control (QC) sample was prepared by mixing equal parts of each sample in order to check UPLC-QToF performance throughout the experiment. MassLynx v4.1 (Waters) was used to manually extrapolate the peak areas relative to the tryptamine and serotonin signals and the corresponding deuterated commercially authentic standards. Peak extrapolation was based on the following *m/z* values, previously chosen after analysis of the commercial standards and that corresponded to the highest in-source generated fragments detected in positive ionization mode: 160.0777 for serotonin and 164.1009 for d_4_-serotonin; 144.0863 for tryptamine and 148.1061 for d_4_-tryptamine. The concentrations of endogenous tryptamine and serotonin in the samples were determined from the area ratios of endogenous to corresponding stable isotope-labeled standards. Previously, to assess the linearity of the method, two calibration curves (*R*^2^ = 0.99) were built by analyzing different solutions with increasing amounts (1–1,000 pg) of d_4_-tryptamine and d_4_-serotonin.

### Gene expression analysis by qRT-PCR

Total RNA was extracted and cDNA synthesized as reported above. A 1:10 dilution of the cDNA solution was used for qRT-PCR analysis to quantify the levels of *SlTDC1*, *SlTDC2*, *SlTDC3* and *SlT5H* mRNA (primers are reported in Supplementary Table 4). A 25-μL reaction was performed in triplicate for each sample in 96-well plates using the GoTaq qPCR Master Mix (Promega, Madison, United States) according to manufacturer’s instructions. A cDNA pool representing all samples was also tested to normalize expression data across plates. The qRT-PCR was performed in a StepOne Plus thermocycler (Applied Biosystems, Foster City, United States) setting the following cycling conditions: initial denaturation at 95°C for 2 min followed by 40 cycles of denaturation at 95°C for 15 s, annealing at 60°C for 30 s and extension at 60°C for 30 s. The amplification process was followed by melting curve analysis (55–95°C) with temperature increments of 0.3°C every 15 s. Raw data were processed with LinRegPCR software to determine baseline and threshold cycles (Ct). Transcript levels were expressed as mean normalized expression (Muller et al., 2002) by Ct comparison with the *SlCAC* calibrator gene (*Solyc08g006960.2.1*; González-Aguilera et al., 2016). Gene expression and metabolomic data were integrated by evaluating the Pearson correlation coefficient in Microsoft Excel (Microsoft, Redmond, United States).

## Results

### Identification and *in silico* analysis of tomato TDC candidates

Several tomato TDC orthologs (SlTDCs) have been predicted and one of them, SlTDC1 (*Solyc07g054860*), was recently shown to produce tryptamine (Tsunoda et al., 2021). We used the amino acid sequences of well-characterized plant TDCs for homology-based screening of the Sol Genomics Network database (SGNdb) to further identify three tomato proteins characterized by high percentage identity scores (Table 1) that we annotated as SlTDC2 (*Solyc07g054280*), SlTDC3 (*Solyc09g064430*) and SlTDC4 (*Solyc03g044120*).

**Table 1:** Properties of the tomato TDC candidates and their relationship with other plant TDCs.

		Putative tomato TDCs			Functionally characterized plant TDCs					
	aa	SlTDC2	SlTDC3	SlTDC4	CrTDC	OsTDC1	OsTDC2	OsTDC3	PepTDC1	PepTDC2
SlTDC1	504	**89.3**	55.3	54.4	68.3	51.9	52.7	53.0	**86.2**	55.4
SlTDC2	504	–	55.8	54.0	66.1	50.7	53.5	51.0	**84.0**	55.4
SlTDC3	468	–	–	52.8	51.0	45.3	68.8	46.1	53.0	**90.4**
SlTDC4	476	–	–	–	51.3	43.8	50.6	43.3	53.5	51.8

The column labeled “aa” shows the number of amino acid residues in each tomato TDC candidate. Other columns show percentage identity scores among the tomato TDCs and other functionally characterized plant. TDCs: CrTDC from *Catharantus roseus* (De Luca et al., 1989), OsTDCs from *Oryza sativa* (Kang et al., 2007a) and PepTDCs from *C. annuum* (Park et al., 2009). Bold entries show top-scoring matches.

The alignment of SlTDCs candidates (Supplementary Figure 1) with other functionally characterized plant TDCs (Supplementary Table 1) revealed the presence of many conserved regions necessary to the decarboxylation reaction including the sites for substrate selectivity and cofactor (pyridoxal-5′-phosphate, PLP) binding (Torrens-Spence et al., 2020; Zhou et al., 2020). The histidine and tyrosine residues in the first and second putative catalytic sites are considered key requirements for l-tryptophan decarboxylation, and are highly conserved in SlTDC1, SlTDC2 and SlTDC3. Contrarily, the tyrosine was replaced by phenylalanine in SlTDC4, in which, moreover, the PLP-binding site was completely missing and the glycine in the putative substrate selection site was replaced with serine. Importantly, glycine at this site facilitates specific binding to indole structures whereas serine binds phenyl amino acids, therefore being necessary to distinguish between tryptophan and tyrosine substrates.

To investigate the phylogenetic relationships between the three putative SlTDCs, SlTDC1 and other plant AADCs, we constructed a phylogenetic tree (Supplementary Figure 2) including the known functionally annotated plant TDCs and tyrosine decarboxylases (TyDCs). SlTDC1 and SlTDC2 clustered in the exclusive TDC family clade, showing close relationships with TDCs from other solanaceous species, whereas SlTDC3 and SlTDC4 did not. SlTDC3 clustered with PepTDC2 and OsTDC2, but this group was closer to functionally characterized TyDCs than other TDCs and was the only group of functionally characterized TDCs excluded from the TDC clade. SlTDC4 clustered in another TyDC clade, close to TyDCs from *Petroselinum crispum* and *Papaver somniferum*.

### Isolation and structural characterization of TDC genes from *Solanum lycopersicum* cv. Micro-Tom

The *SlTDC1*, *SlTDC2* and *SlTDC3* CDS that we cloned (Supplementary Information 1) from different Micro-Tom organs were found to be identical to those deposited for cv. Heinz 1706 in SGNdb (Table 2). In contrast, the amplification of *SlTDC4* from Micro-Tom total cDNA did not yield a PCR product, suggesting this gene is not expressed in Micro-Tom plants under standard growing conditions. This hypothesis was supported by the negligible expression levels of SlTDC4 reported in Heinz 1706 microarray (Consortium, 2012) and Micro-Tom RNA-Seq (TomExpress) expression datasets (Supplementary Figure 3).^4^ The *SlTDC4* sequence was therefore amplified from Micro-Tom genomic DNA (Supplementary Information 2).

**Table 2:** Structural features of the *SlTDC* genes cloned from Micro-Tom cDNA or genomic DNA.

Gene	Source	Organ	Putative CDS length (bp)	Identity (%)^1^	Putative protein length (aa)
*SlTDC1*	total cDNA	mature-green fruit	1,515	100	504
*SlTDC2*	total cDNA	flowers	1,515	100	504
*SlTDC3*	total cDNA	leaves	1,407	100	468
*SlTDC4*	genomic DNA	leaves	1,431	99.6^†^	476
*SlT5H*	total cDNA	mature-green fruit	1,588	99.9	495

1 The percentage identity refers to the comparison of the Micro-Tom sequenced CDS and the sequences in SGNdb.

† In the case of *SlTDC4*, the percentage identity refers to the comparison between the cloned Micro-Tom *SlTDC4* and the *Solyc03g044120* gene in SGNdb.

### Functional characterization of Micro-Tom TDCs by untargeted metabolomics

**Figure 1:**
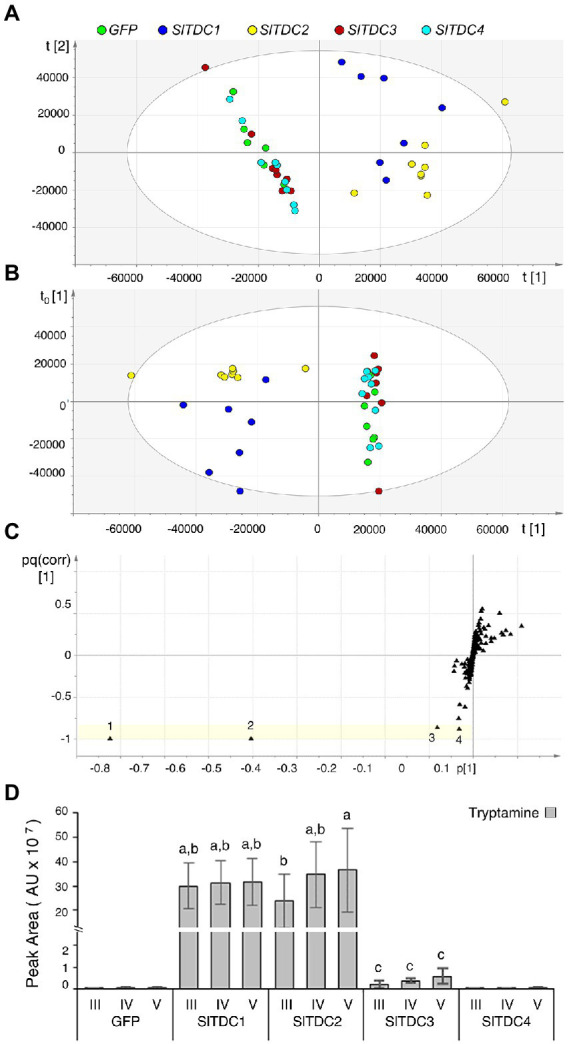
Multivariate statistical analysis of the untargeted metabolomics dataset (positive ionization) following the HPLC-ESI-MS analysis of *Nicotiana benthamiana* plants transiently expressing individual *SlTDC* genes or GFP as a negative control. **(A)** PCA score scatter plot. **(B)** OPLS-DA score scatter plot. **(C)** OPLS-DA S-loading plot, with each metabolite represented by a black triangle. The yellow area highlights metabolites characteristic of the *SlTDC1/SlTDC2* cluster but not the *GFP*/*SlTDC3*/*SlTDC4* cluster: 1 and 2 represent molecular and fragment ions of tryptamine (161 and 144 *m/z*, respectively), 3 is a molecular ion of serotonin (177 *m/z*), and 4 is an unidentified metabolite (177 *m/z*). **(D)** Levels of tryptamine in the infiltrated leaves. AU = arbitrary unit. Data are means ± standard deviations (*n* = 3). Different letters on bars indicate that means are significantly different according to *post-hoc* Tukey-ANOVA (*p* < 0.05).

To assess the function of the Micro-Tom SlTDC candidates, we developed an *in vivo* functional assay based on transient expression of the *SlTDC* genes in *Nicotiana benthamiana,* which does not produce detectable levels of tryptamine or serotonin in its aerial organs. The constructs harboring the CDS of *SlTDC1*, *SlTDC2*, *SlTDC3* and *SlTDC4* under the control of the strong constitutive cauliflower mosaic virus 35S (CaMV35S) promoter were introduced *via Agrobacterium tumefaciens* into *N. benthamiana* plants. Leaf methanol extracts were analyzed by HPLC-ESI-MS and two datasets corresponding to negative and positive ionization were created for multivariate statistical analysis. PCA and OPLS-DA of the negative data matrix revealed no clustering among the samples. In contrast, PCA (Figure 1A) and corresponding OPLS-DA (Figure 1B) of the positive data matrix revealed two distinct groups, with the *SlTDC1* and *SlTDC2* infiltrated plants in one cluster and the *SlTDC3* and *SlTDC4* infiltrated plants in the other, along with the negative control expressing green fluorescent protein (GFP). OPLS-DA highlighted specific metabolites that were highly correlated with each group (Figure 1C). The in-source fragment and molecular ion of tryptamine were the metabolites that correlated best with the *SlTDC1* and *SlTDC2* infiltrated plants (Figure 1D) followed by serotonin and an unidentified metabolite (Supplementary Figure 4). Conversely, the clustering of the *SlTDC3* and *SlTDC4* infiltrated plants with the GFP control indicated similar metabolic profiles in all three cases. Even so, the *SlTDC3* infiltrated plants accumulated small amounts of tryptamine and serotonin, whereas neither of these metabolites was detected at significant levels in leaves transiently expressing *SlTDC4* (Figure 1D; Supplementary Figure 4), suggesting that this gene does not function as a TDC (further evidence is presented in Supplementary Information 3).

### Identification and functional characterization of a putative Micro-Tom T5H

The high levels of serotonin detected in tomato, as reported by recent literature, suggest the existence of a SlT5H that converts tryptamine to serotonin. We therefore sought candidate tomato *T5H* genes by using the previously characterized OsT5H (Fujiwara et al., 2010) as a blastp search query against SGNdb. This recovered Solyc09g014900.2 (SlT5H), which shares 55.3% identity with OsT5H and is annotated as a cytochrome P450 monooxygenase predicted by Predotar to be localized to the ER. Repeating the strategy deployed for TDC (see Supplementary Information 1), we used the *SlT5H* CDS as a blastn query against the KaFTom database and recovered a sequence with 99.93% identity (LEFL1021DE05). The sequences were translated *in silico* and the alignment revealed only one substitution. The *SlT5H* CDS deposited in SGNdb was cloned from cDNA prepared from mature-green Micro-Tom fruits (Table 2) and transferred to vector pK7WG2 for functional characterization in *N. benthamiana* plants.

**Figure 2:**
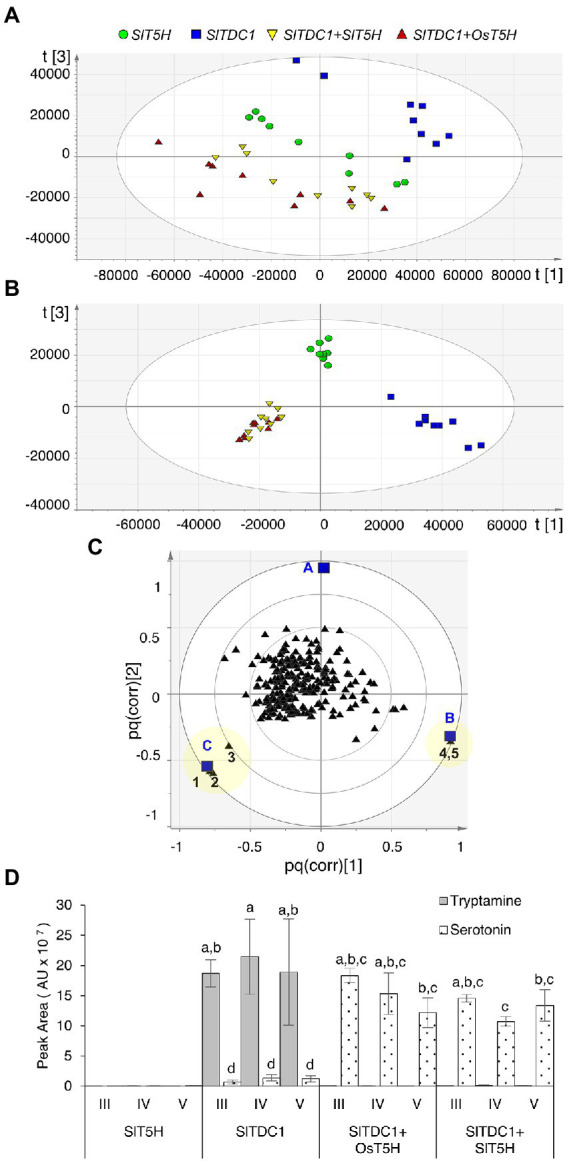
Multivariate statistical analysis of the untargeted metabolomics dataset (positive ionization) following the HPLC-ESI-MS analysis of *Nicotiana benthamiana* plants transiently expressing *SlTDC1* and *T5H* genes. **(A)** PCA score scatter plot. **(B)** O2PLS-DA score scatter plot. **(C)** O2PLS-DA correlation loading plot, with each metabolite represented by a black triangle and classes by blue squares (A = *SlT5H*, B = *SlTDC1*, and C = *SlTDC1* + *OsT5H* and *SlTDC1* + *SlT5H*). The yellow area highlights metabolites characteristic of each class: 1 and 2 represent molecular and fragment ions of serotonin (177 and 160 *m/z*, respectively), 3 is an unidentified metabolite (222 *m/z*), and 4 and 5 are molecular and fragment ions of tryptamine (161 and 144 *m/z*, respectively). **(D)** Levels of tryptamine and serotonin. AU = arbitrary unit. Data are means ± standard deviations (*n* = 3). Different letters on bars indicate that means are significantly different according to post-hoc Tukey-ANOVA (p < 0.05).

Plants were infiltrated simultaneously with pK7WG2.*SlTDC1* and pK7WG2.*SlT5H* whereas plants infiltrated with either of the individual genes were considered as negative controls. Plants infiltrated with *SlTDC1* and *OsT5H* (the *bona fide* rice *T5H* gene) were used as positive controls. Methanolic extracts were analyzed by HPLC-ESI-MS and the untargeted metabolomics datasets were explored by PCA and O2PLS-DA as above. PCA of the positive data matrix resolved three distinct clusters: plants expressing *SlTDC1*; plants expressing *SlT5H*; and plants co-expressing *SlTDC1* and *OsT5H* or *SlTDC1* and *SlT5H* (Figure 2A). The O2PLS-DA score scatter plot (Figure 2B) confirmed the PCA clustering and identified molecules that correlated with each of the clusters (Figure 2C). Serotonin correlated solely with the *SlTDC1* + *OsT5H* and *SlTDC1* + *SlT5H* plants (Figure 2D), along with an unidentified metabolite (222 *m/z*) that was present at much lower levels (Supplementary Figure 5). Our data confirmed that SlT5H converts tryptamine to serotonin and can thus be considered as a *bona fide* T5H. As above, analysis of the negative data matrix revealed no clustering among the samples.

### Targeted metabolomics reveals the dynamic distribution of tryptamine and serotonin in different tomato organs during development

**Figure 3:**
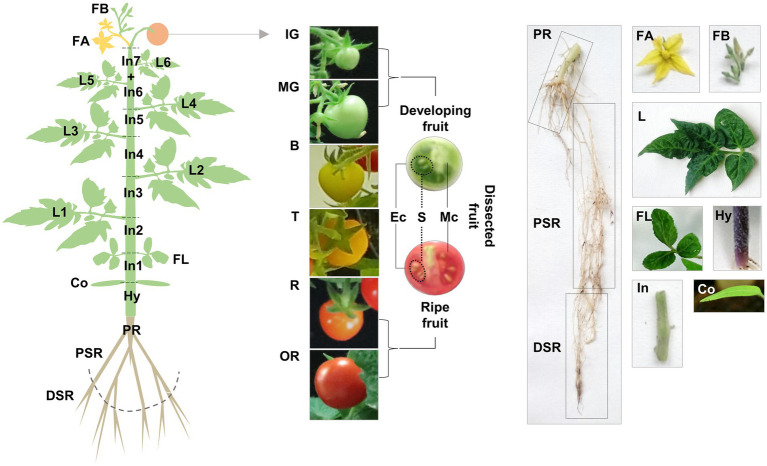
Schematic representation of our sampling strategy for gene expression profiling and targeted metabolomics. Vegetative organs: roots (PR, primary root; PSR, proximal secondary root; DSR, distal secondary root), stem (Hy, hypocotyl; In, internode), cotyledons (Co), leaves (FL, first leaves; L, true leaves). Reproductive organs: flowers (FB, flower buds; FA, flowers at anthesis), fruit (IG, immature-green; MG, mature-green; B, breaker; T, late breaker/turning; R, ripe; OR, over-ripe), dissected fruit (Ec, exocarp; Mc, mesocarp; S, seeds).

Having discovered multiple *TDC* genes in tomato, we explored their functional diversification by gene expression profiling and the targeted analysis of tryptamine and serotonin in various Micro-Tom organs collected at different stages (Figure 3).

**Figure 4:**
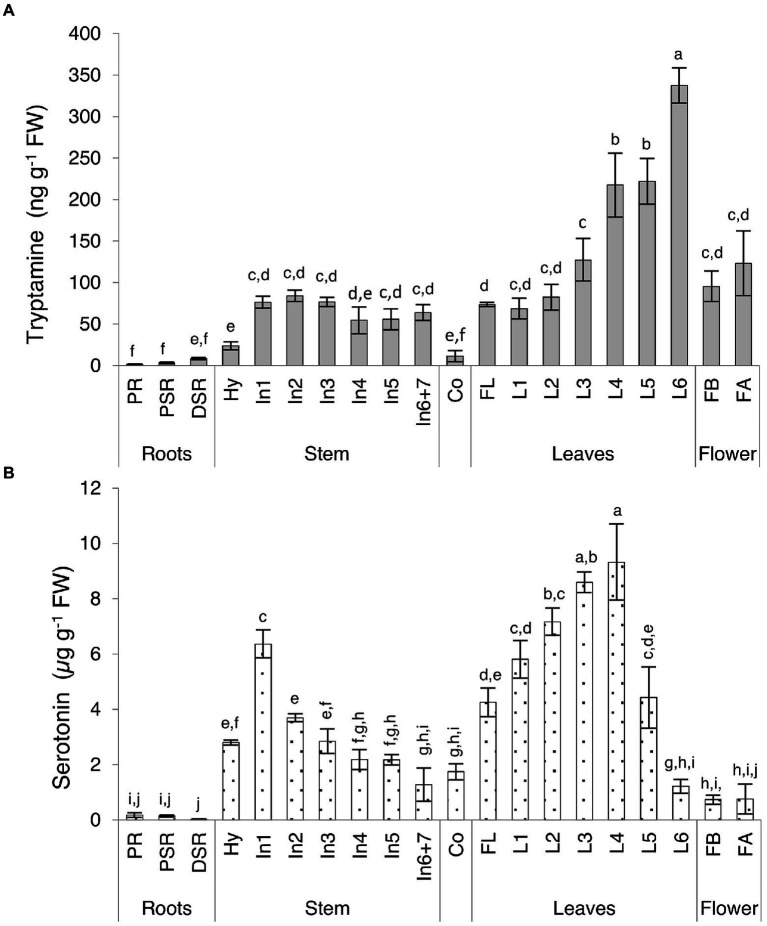
Quantities of **(A)** tryptamine and **(B)** serotonin detected in Micro-Tom vegetative organs and flowers by UPLC-ESI-MS analysis expressed as **(A)** ng g^−1^ FW or **(B)** μg g^−1^ FW. Data are means ± SD (*n* = 3). Different letters on bars indicate that means are significantly different according to post-hoc Tukey-ANOVA (*p* < 0.05). The abbreviations and the position of the samples are explained in Figure 3.

We observed major differences in the levels of both indolamines in the vegetative organs, revealing distinct accumulation patterns (Supplementary Table 5; Figure 4). Serotonin levels in some organs were up to 150-fold higher than tryptamine levels, with the total concentration ranging from 27.19 to 9322.02 ng g^−1^ FW for serotonin and 1.60 to 337.60 ng g^−1^ FW for tryptamine. The lowest levels of both compounds were detected in the roots, with opposing profiles (tryptamine increasing, serotonin decreasing) moving from the primary embryonal root to the distal part of the secondary roots. The highest levels of both compounds were detected in the leaves, with distinct trends in the older basal leaves and the younger ones close to the shoot apex. Tryptamine accumulation increased in a gradient along the longitudinal axis of the plant, from the first leaves to the leaves of the last upper node (Figure 4A), whereas serotonin accumulation increased until the leaves of the fourth node and then sharply dropped in the leaves of the fifth and sixth nodes (Figure 4B). Interestingly, a serotonin gradient opposite to that observed in the leaves was detected in the stem, with the levels declining from the first to the last internodes (Figure 4B). In flowers, serotonin was approximately six-fold more abundant than tryptamine, but we observed no significant change in abundance between the flower bud stage and fully-open flower stage.

**Figure 5:**
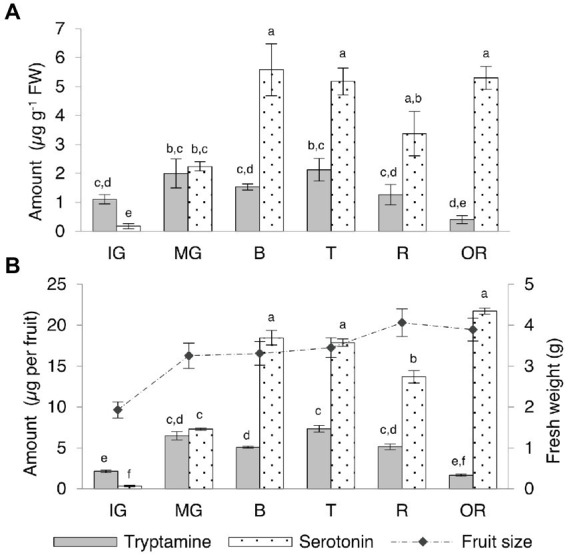
Quantities of tryptamine and serotonin detected by UPLC-ESI-MS in Micro-Tom fruits at different developmental and ripening stages expressed as **(A)** μg g^−1^ FW and as **(B)** μg per whole berry. Data are means ± SD (*n* = 3). Different letters on bars indicate that means are significantly different according to *post-hoc* Tukey-ANOVA (*p* < 0.05). The abbreviations and the position of the samples are explained in Figure 3.

Tryptamine and serotonin reached similar levels in the Micro-Tom fruit, in contrast to the other organs (Supplementary Table 6; Figure 5A). Tryptamine was 10-fold more abundant than serotonin at the immature-green stage and levels slightly increased at the mature-green stage in concert with rapid fruit growth, before leveling off during ripening and declining slightly in the over-ripe fruit. Serotonin levels increased rapidly between the immature and mature-green fruit stages, becoming more or less equal to tryptamine in the mature-green fruit and then continuing to increase until the breaker stage (onset of ripening), then leveling off and remaining fairly constant until senescence. By normalizing the quantity of tryptamine and serotonin to the average fresh weight of the fruits at each stage, we were able to express the indolamine content per whole fruit (Supplementary Table 6; Figure 5B). The fully-ripe berry fruit contained ~5 μg of tryptamine and ~ 14 μg of serotonin.

**Figure 6:**
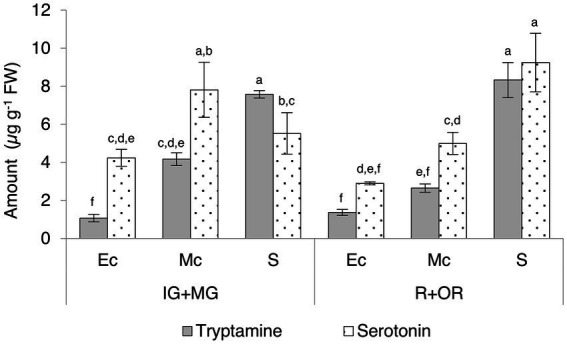
Quantities of tryptamine and serotonin detected by UPLC-ESI-MS in the skin (exocarp, Ec), flesh (mesocarp, Mc) and seeds (S) of unripe (IG + MG) and ripe (R + OR) Micro-Tom fruits, expressed as μg g^−1^ FW. Data are means ± SD (*n* = 3). Different letters on bars indicate that means are significantly different according to *post-hoc* Tukey-ANOVA (*p* < 0.05). The abbreviations and the position of the samples are explained in Figure 3.

We also divided the unripe (immature-green and mature-green) and fully-ripe (ripe and over-ripe) berries into different tissues (exocarp, mesocarp and seeds) for further investigation (Supplementary Table 7; Figure 6). Seeds contained the highest levels of tryptamine and serotonin and a slight increase in serotonin was observed as the fruit ripened. Intermediate and high levels of tryptamine and serotonin, respectively, in the mesocarp were observed to decrease during the ripening process, but serotonin was consistently twice as abundant as tryptamine. The lowest levels of both compounds were detected in the skin, but serotonin was four-fold more abundant than tryptamine at the unripe stage and twice as abundant in the ripe fruit.

### Expression profiles of SlTDCS and SlT5H

**Figure 7:**
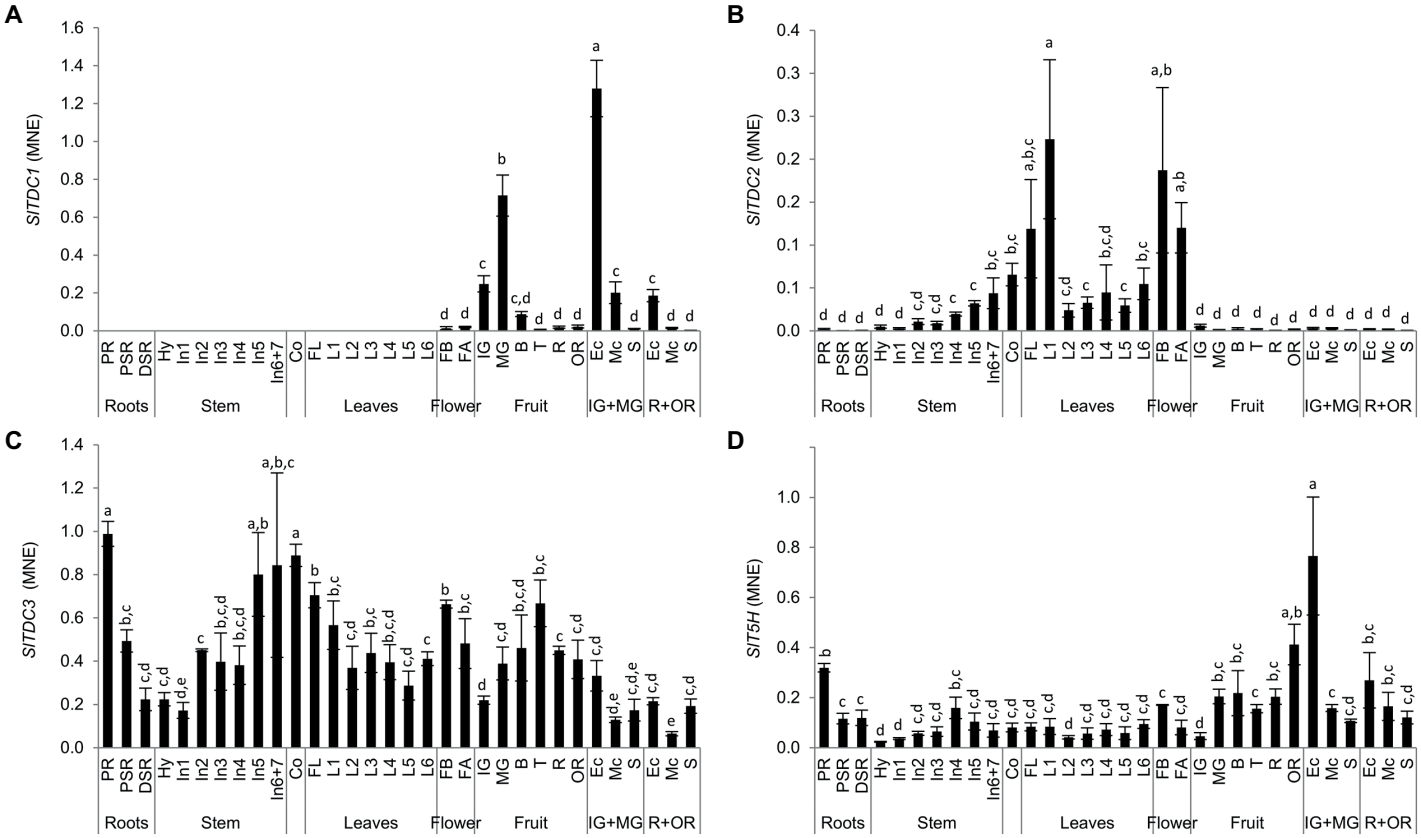
Analysis of **(A)** *SlTDC1*, **(B)** *SlTDC2*, **(C)** *SlTDC3* and **(D)** *SlT5H* expression in Micro-Tom organs by qRT-PCR. Data are means ± SD (*n* = 3). MNE = Mean normalized expression. Different letters on bars indicate that means are significantly different according to post-hoc Tukey-ANOVA (*p* < 0.05). The abbreviations and the position of the samples are explained in Figure 3.

RNA extracted from the same samples used for targeted metabolomics was used to determine the expression profiles of the *SlTDC* and *SlT5H* genes by quantitative real-time PCR (qRT-PCR). *SlTDC1* was expressed solely in the reproductive organs (Figure 7A), with low levels of mRNA in the flowers and immature-green berry, but a burst of expression toward the end of the mature-green stage. This peak was followed by a drop from the breaker stage to the over-ripe fruit. The highest levels of *SlTDC1* mRNA were present in the exocarp of the developing berry and, to a lesser extent, in the ripe fruit. The transcript levels in the seeds remained remarkably low.

In contrast, *SlTDC2* was expressed mainly in the aerial vegetative organs and flowers with minimal levels in the primary root and fruit (Figure 7B). Interestingly, we observed an *SlTDC2* expression gradient in the stem, with the lowest levels in the hypocotyl and basal internodes and the highest levels in the last internodes. *SlTDC2* was expressed strongly in the flowers, cotyledons and oldest leaves of the plant, followed by a sharp decrease immediately after the first node.

In contrast to the restricted profiles of *SlTDC1* and *SlTDC2*, *SlTDC3* was expressed in all plant organs (Figure 7C). However, we still observed opposing expression gradients in the stems and leaves, with increasing transcript levels from the hypocotyl to the upper internodes but higher levels in older than younger leaves. *SlTDC3* was strongly expressed in the primary root and lower but still significant mRNA levels were detected in the secondary roots. *SlTDC3* expression also increased in fruits following the end of berry development and peaked at the turning stage, complementing the low levels of *SlTDC1* mRNA at this stage. *SlTDC3* mRNA levels were higher in the skin and seeds of ripe berries than the flesh.

*As SlTDC3, also SlT5H* was ubiquitously expressed (Figure 7D). Among the vegetative organs, the highest *SlT5H* mRNA levels were observed in the primary root. A slight increase in expression was observed in the stem from the hypocotyl to the fourth internode, followed by a decrease in the younger stem portion, thus mirroring the distribution of serotonin in the leaves. *SlT5H* mRNA levels were similar in all leaves and the cotyledons, but were slightly higher in the flower buds than in the flower at anthesis. *SlT5H* was expressed throughout fruit development and ripening, peaking at the mature-green stage and then remaining constant until the over-ripe stage, when a further increase was observed. Notably, as observed for *SlTDC1*, the highest levels of *SlT5H* mRNA levels were detected in the exocarp of unripe berries.

**Figure 8:**
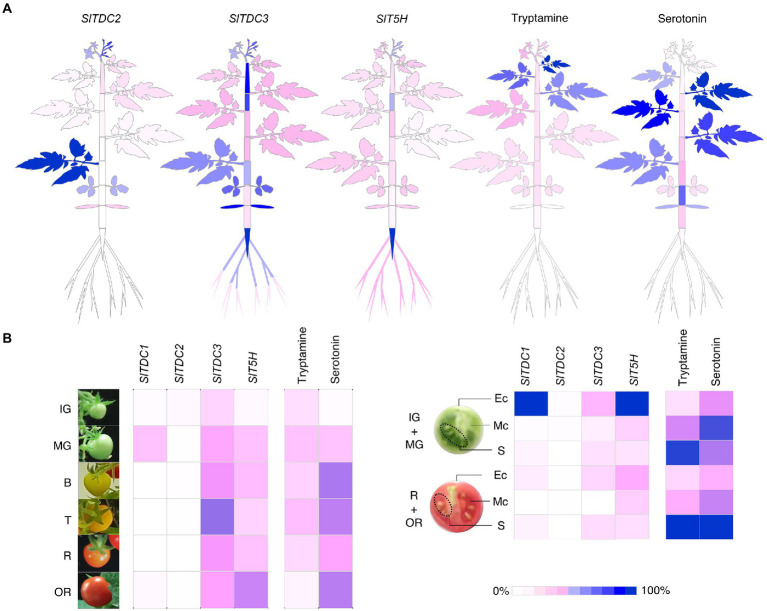
Heat maps representing the percentage relative expression of *SlTDCs and SlT5H* genes and the accumulation of tryptamine and serotonin in Micro-Tom plants **(A)** and fruits **(B)**. Expression levels are shown as percentages relative to the organ with the highest expression (100%). Each organ and stage/tissue identified in this heat map refers to the sampling design in Figure 3.

To better represent these data, we generated heat map atlases of gene expression and indolamine levels in the vegetative organs (Figure 8A) and developing/ripening fruit (Figure 8B).

### The relationship between indolamine accumulation and TDC and T5H gene expression

To gain insight into the relationship between indolamine accumulation and *TDC* and *T5H* gene expression in tomato, we looked for linear correlations between the gene expression profiles and metabolites by calculating Pearson correlation coefficients (PCCs; Supplementary Figure 6). This revealed a positive correlation between *SlTDC3* and *SlT5H* expression in the roots and the production of serotonin, and a negative correlation between these three factors and the accumulation of tryptamine, suggesting that *SlTDC3* and *SlT5H* expression results in the depletion of tryptamine in the roots and its conversion to serotonin. In the basal leaves (from the cotyledons to the leaves of the third node), we observed a positive correlation between tryptamine and serotonin levels and between *SlT5H* and *SlTDC2* expression. The levels of both indolamines increased along the shoot axis toward the apex although the tryptamine gradient was shallower than that of serotonin. These profiles showed a negative correlation with the expression of *SlTDC3*. On the other hand, in the upper leaves (between the fourth and sixth nodes), we observed a strong negative correlation between tryptamine and serotonin levels, reflecting the gradient of serotonin in leaves up to the fourth node followed by a rapid drop-off in leaves next to the shoot apex. In these organs, we also observed a positive correlation between *SlTDC3* and *SlT5H* expression that appeared unrelated to indolamine accumulation. No clear relationships were observed in the fruit until PCCs were calculated by distinguishing the phases of fruit development and ripening. In unripe berries (analysis based on IG, MG and B samples), *SlTDC1* expression showed strong positive correlation with the accumulation of tryptamine whereas *SlTDC3* and *SlT5H* expression correlated with the accumulation of serotonin. During the ripening phase (analysis based on T, R and OR samples), tryptamine accumulation was strongly negatively correlated with *SlTDC1* expression, which was near zero in all but the OR samples, and weakly positively correlated with the expression of *SlTDC3*. Tryptamine also showed a strong negative correlation with *SlT5H* expression. Interestingly, *SlT5H* expression was not positively correlated with serotonin.

## Discussion

### Homology analysis of SlTDC candidates and the need for direct functional characterization

In this work, we have provided new evidence for the functional characterization of tomato genes that were recently proposed to be involved in tryptamine and serotonin production.

Two *in silico* studies suggested the existence of two up to five TDCs in tomato (Hano et al., 2017; Pang et al., 2018), and the function of one isoform (SlTDC1) has already been assessed in this species (Tsunoda et al., 2021). By using homology-based screening we identified three SlTDCs candidates characterized by high percentage identities to SlTDC1 and other plant TDCs. With the exception of SlTDC4, all SlTDCs were found to share key residues required for PLP binding, tryptophan selection and catalytic activity (Torrens-Spence et al., 2020; Zhou et al., 2020). Phylogenetic analysis revealed that SlTDC1 and SlTDC2 clustered with other solanaceous TDCs whereas SlTDC4 was phylogenetically closer to functionally characterized TyDCs. However, these automatic homology-based classifications are not always reliable due to the similarity between TDCs and TyDCs (Abu-Zaitoon, 2014). For example, SlTDC3 clustered with the characterized TDCs from rice and bell pepper, which show closer phylogenetic proximity to TyDCs than to other plant TDCs. Homology-based screening is thus a useful but insufficient tool to assign gene functions. We therefore developed an *in planta* assay system for direct functional characterization of the SlTDC candidates to confirm their TDC activity.

### Three TDC genes and one SlT5H gene are responsible for tryptamine and serotonin biosynthesis in tomato

The transient expression of SlTDCs in *N. benthamiana*, a solanaceous species that does not accumulate detectable levels of tryptamine or serotonin in its leaves, revealed that SlTDC1, SlTDC2 and SlTDC3 were *bona fide* TDCs because the infiltrated plants accumulated tryptamine. Interestingly, the lower tryptamine levels observed for *SlTDC3* suggest lower enzyme activity or lower substrate affinity for this candidate. Although substrates other than tryptophan might be preferred by SlTDC3, we detected no metabolites exclusive to the *SlTDC3* infiltrated plants, providing strong evidence for a specific TDC function.

Tomato plants accumulate serotonin in a spatiotemporally regulated manner suggesting the presence of an enzyme that synthesizes serotonin from tryptamine (Islam et al., 2016; Hano et al., 2017) orthologous to the *OsT5H* gene previously discovered in rice (Fujiwara et al., 2010). Both the *OsT5H* gene and its putative tomato ortholog represent the CYP71 group of cytochrome P450 monooxygenases. Through untargeted metabolomics analysis we revealed the accumulation of serotonin in *N. benthamiana* plants infiltrated with both *SlTDC1* and *SlT5H*, thus confirming the function of SlT5H. Moreover, tryptamine was detected in only trace amounts as a residual substrate in these plants, indicating a high rate of conversion by SlT5H.

### Tryptamine and serotonin show distinct spatiotemporal profiles in tomato plants and fruits

We investigated the spatiotemporal distribution of tryptamine and serotonin in Micro-Tom fruits as well as the vegetative and reproductive organs from early development to senescence, thus adding more details to the information already available for this species (Udenfriend et al., 1959; West, 1959; Feldman and Lee, 1985; Hano et al., 2017). Targeted UPLC-ESI-MS analysis revealed the organ-specific accumulation of these compounds in whole plants and their tissue-specific accumulation in fruits. Tryptamine accumulated to high levels only in the reproductive organs (up to ~8.33 μg g^−1^ FW in the seeds) whereas, in accordance with recent literature (Negri et al., 2021), levels were much lower in the rest of the plant (up to ~0.34 μg g^−1^ FW in younger leaves). In contrast, yet as commonly observed in many plant species (Erland et al., 2016), serotonin also accumulated to high levels in some vegetative organs (up to ~9.32 μg g^−1^ FW) hinting that these indolamines serve different tissue-specific purposes. The large quantities of serotonin detected in all plant tissues, and the accumulation of tryptamine in the flowers, fruit and seeds, suggest that neither indolamine is a mere intermediate but also an end-product with a specific biological function.

### Indolamine gradients along the growth axis hint at biological roles in the vegetative organs

The measurement of tryptamine and serotonin levels in leaves at each internode along the longitudinal axis of Micro-Tom plants revealed a gradient of both molecules. Serotonin increased from low levels at the basal nodes, peaking at the fourth node and then sharply declining in the leaves close to the terminal bud, confirming the gradient is not just a consequence of dilution due to the expansion of older leaves. In contrast, tryptamine levels increased continuously and were highest in the top two leaves. Several reports suggest that tryptamine interferes with insect reproduction by inhibiting oviposition, feeding and/or larval development (Thomas et al., 1998, 1999; Gill et al., 2003). The higher tryptamine content in young tomato leaves may serve as a deterrent to protect these developing photosynthetic organs, which are more attractive to insects. Interestingly, although tryptamine levels were constant along the stem, we observed a gradient of serotonin with opposite polarity to the gradient in leaves. Concentration gradients in the stem have already been reported for phytohormones including auxin (Kojima et al., 2002), abscisic acid (Everat-Bourbouloux and Charnay, 1982) and cytokinins (Eklöf et al., 1996), yet at much lower levels than serotonin (10–0.1 ng g^−1^ FW).

### Dynamic indolamine profiles during fruit development and ripening suggest additional functions

Many berries accumulate tryptamine and serotonin at similar or higher levels than tomato (Erland et al., 2016; Islam et al., 2016) with some fruits, including banana, kiwifruit, grape and pineapple, showing no specific developmental trends in indolamine levels (Udenfriend et al., 1959; Foy and Parratt, 1960; Murch et al., 2010; Commisso et al., 2019). We found that serotonin and tryptamine accumulate in developing Micro-Tom fruits, with tryptamine reaching a plateau at the mature-green stage, just before the onset of ripening, and serotonin reaching a plateau at the breaker stage. Evidence that the indolamines do not accumulate merely as a consequence of development but are involved in its regulation comes from the overexpression of *SlTDC1* in tomato, which produced serotonin-rich fruits with a shorter transition to the breaker stage compared to wild-type plants (Tsunoda et al., 2021). The authors proposed an interaction between serotonin and the ethylene pathway that regulates berry ripening in tomato, but further investigation is required.

We also measured the accumulation of indolamines in different parts of the unripe and ripe berry. The unripe and ripe seeds accumulated large amounts of tryptamine and serotonin whereas moderate levels were detected in the mesocarp and low levels in the exocarp. This partially agrees with previous studies (Feldman and Lee, 1985; Hano et al., 2017) with differences, in this case, attributed to the different cultivars analyzed or even to different growing practices, which have been recently demonstrated to affect indolamine content in tomato (Lee et al., 2021).

### Dynamic SlTDC and SlT5H expression profiles explain spatiotemporal variations in tryptamine and serotonin levels and suggest their involvement in distinct biological functions

The gene expression profiling that we performed though our sophisticated experimental design revealed complementary expression trends among the *SlTDC* genes to coordinate tryptamine biosynthesis with an organ-specific distribution. *SlTDC1* was expressed only during the reproductive phase. Conversely, *SlTDC2* was strongly expressed in the aerial vegetative organs and flowers but not in the fruit. *SlTDC3* was expressed at different levels in all organs, and was the only *SlTDC* gene active in the roots. It must be considered that SlTDC1 and SlTDC2 were much more efficient than SlTDC3 in the conversion of tryptophan to tryptamine in our heterologous system. Therefore, even the low levels of *SlTDC1* and *SlTDC2* expression in the flowers, shoots and fruit tissues may be more relevant to tryptamine production than the expression profiles suggest, whereas the ubiquitously expressed but less active SlTDC3 may play a different role in determining tryptamine levels. In this context, tryptamine levels in tomato appear to be finely regulated by different mechanisms, suggesting that it is not merely a metabolic intermediate for the production of serotonin, melatonin and their derivatives but may also perform multiple biological functions.

In plants with at least two *TDC* genes (e.g., bell pepper and *Camptotheca acuminata*), one of the genes tends to be responsive to stress (López-Meyer and Nessler, 1997; Park et al., 2009). In rice, very low level constitutive *TDC* gene expression is boosted by nutrient depletion, which induces senescence in the leaves (Kang et al., 2009). Adding to the previous work on *TDC* genes in tomato (Hano et al., 2017; Tsunoda et al., 2021), we show for the first time that the tomato *TDC* gene family is also regulated in a complex manner, with different paralogs showing spatiotemporally regulated expression profiles.

In contrast to the intricate regulation of *TDC* genes, we found that *SlT5H* was expressed in all Micro-Tom organs, as previously observed in tomato (Hano et al., 2017) and rice (Kang et al., 2007b). T5H activity is highest in the roots of both species, but only in rice does this correspond to the accumulation of large amounts of serotonin.

### The linear correlation between indolamine biosynthesis and accumulation supports a model of functional interactions among genes in the serotonin pathway

**Figure 9:**
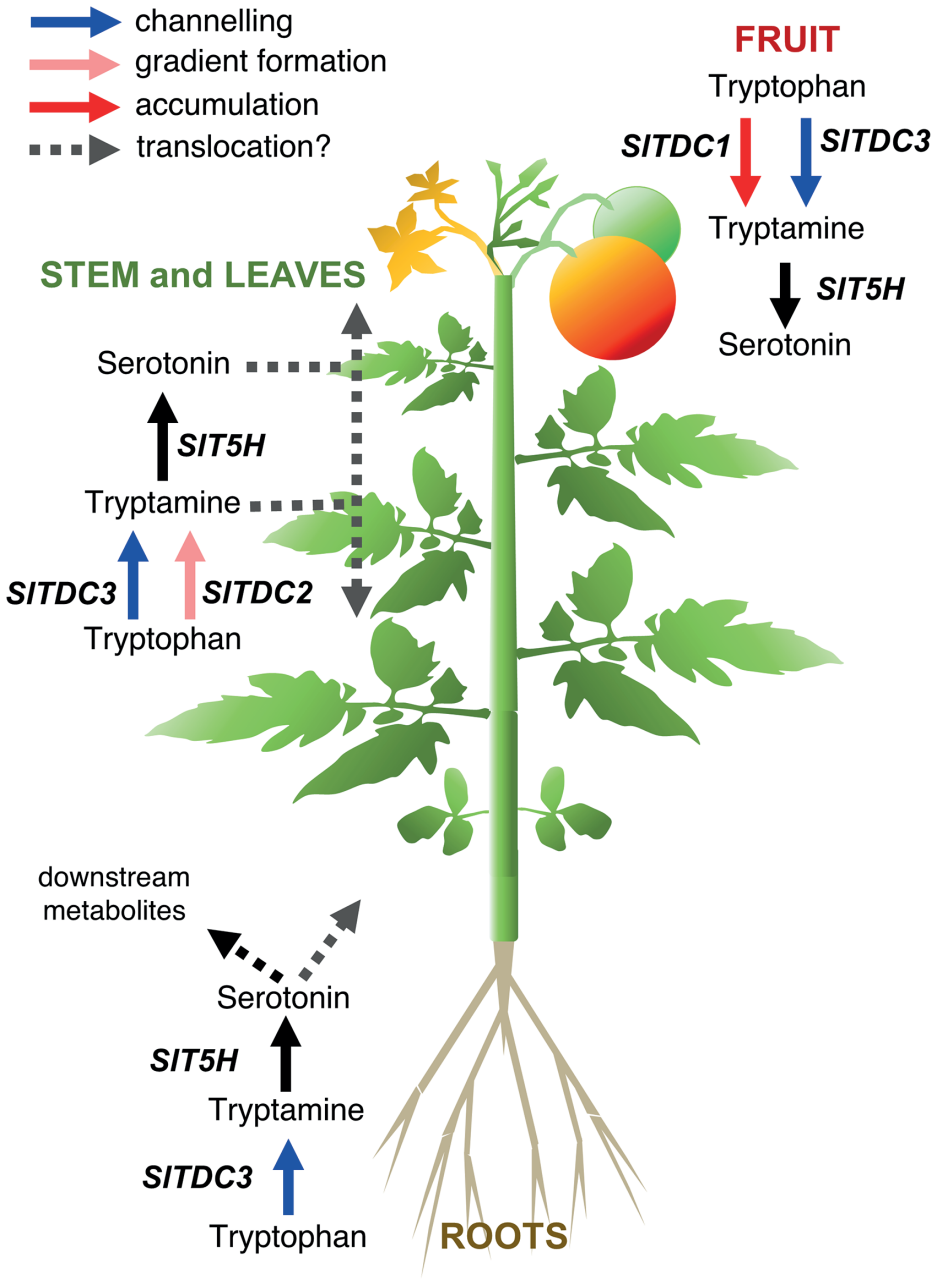
Proposed model for the functional interactions of tomato serotonin pathway genes in different organs of the Micro-Tom plant. Different functions of the *TDC* genes that we hypothesize are represented by arrows with different colors: *SlTDC1* converts tryptophan to tryptamine for accumulation (red arrow) in the immature fruit, *SlTDC3* works in cooperation with *SlT5H* to channel (blue arrows) tryptophan to serotonin in fruit, aerial organs and roots. *SlTDC2* works together with *SlT5H* to generate the indolamine accumulation gradient along the aerial growth axis (pink arrows).

By combining the spatiotemporal gene expression and indolamine accumulation profiles, we were able to build a model (Figure 9) showing the dynamic regulation of indolamine biosynthesis and, in some organs, their channeling into downstream pathways, such as melatonin or feruloyl and coumaroyl derivatives (which should be measured in future studies).

Pearson correlation analysis suggested that *SlTDC3* and *SlT5H* may channel tryptamine into serotonin production in the roots. However, the absence of high serotonin levels in the roots suggests conversion to downstream products or translocation to other parts of the plant.

*SlTDC1* and *SlTDC3* were expressed in the fruit, suggesting that *SlTDC1* might ensures the production of sufficient tryptamine for a specific function during berry development whereas *SlTDC3* and *SlT5H* might work together to regulate the serotonin content. Interestingly, *SlTDC1* and *SlT5H* were expressed strongly in the fruit but the tryptamine and serotonin content was lower than in some vegetative organs with minimal *SlTDC1* and *SlT5H* expression. This may reflect the translocation of these indolamines to the berry flesh or even the seeds, which accumulated the highest levels of both indolamines despite the low gene expression levels.

The relationships between genes and metabolites in the rest of the plant are much more complex given the gradients of gene expression and indolamine accumulation in the leaves and internodes of the stem. *SlTDC2* appears to play a fundamental role in the vegetative aerial organs of Micro-Tom plants, but the expression profile did not precisely match the distribution of tryptamine or serotonin in these organs. Indolamine levels in the root and along the longitudinal axis of the Micro-Tom plant, as well as the expression profiles of the *SlTDC* and *SlT5H* genes, are roughly consistent with the auxin gradients, as reported in Arabidopsis roots (Ljung et al., 2005; Petersson et al., 2009) and the tomato stem (Kojima et al., 2002). The discovery of tryptophan-dependent pathways that lead to auxin biosynthesis suggests the involvement of tryptamine as an intermediate, but this hypothesis is still under debate (Brumos et al., 2014). Mechanisms other than the direct involvement of tryptamine in auxin biosynthesis should also be considered, such as signaling *via* auxin receptors facilitated by the structural similarities between plant indolamines and indole acetic acid (Erland et al., 2015; Mukherjee, 2018; Pelagio-Flores et al., 2018).

In conclusion, the complex expression profiles of the three *TDC* genes therefore suggest unique biological roles despite the conserved catalytic function of the corresponding proteins. Similarly, the dynamic spatiotemporal concentration of the indolamines indicate they play unique and beneficial roles in tomato plants but are not indispensable, given the absence of these metabolites in some plant species.

## Data availability statement

The original contributions presented in the study are included in the article/Supplementary material; further inquiries can be directed to the corresponding authors.

## Author contributions

FG and LA conceived and supervised the research. MC and SN performed the *in silico* analysis, targeted and untargeted metabolomics experiments, analyzed and interpreted data, and wrote the draft article. MC, SN, EF, and CP performed the transient expression experiments. EG and SN performed the gene expression experiments and analyzed and interpreted data. SN integrated data from the gene expression and metabolomics datasets. FG, LA, SN, and MC revised the draft. All authors contributed to the article and approved the submitted version.

## Conflict of interest

The authors declare that the research was conducted in the absence of any commercial or financial relationships that could be construed as a potential conflict of interest.

## Publisher’s note

All claims expressed in this article are solely those of the authors and do not necessarily represent those of their affiliated organizations, or those of the publisher, the editors and the reviewers. Any product that may be evaluated in this article, or claim that may be made by its manufacturer, is not guaranteed or endorsed by the publisher.

## Footnotes

^1^ https://solgenomics.net/tools/blast/

^2^ https://www.ebi.ac.uk/Tools/msa/clustalo/

^3^ http://mzmine.sourceforge.net/

^4^ http://tomexpress.toulouse.inra.fr/
