# Supplementary material for: Indolamine accumulation and TDC/T5H expression profiles reveal the complex and dynamic regulation of serotonin biosynthesis in tomato (Solanum lycopersicum L.)
Source: Front Plant Sci. 2022 Aug 11;13:975434. doi: 10.3389/fpls.2022.975434 (PMC9405198; doi:10.3389/fpls.2022.975434)
Supplement: Supplementary file 1 [file Data_Sheet_1.PDF]

Supplementary Material

Supplementary Figures

Supplementary Figure 1. Partial alignment of the amino acid sequences of candidate SlTDCs and other functionally characterized plant TDCs (Supplementary Table 1). Putative sites for catalytic activity (CA1 and CA2), cofactor binding (CB) and substrate selection (Sus) are shown. Species abbreviations: Ac, *Actinidia chinensis*; Ad, *Actinidia deliciosa*; Av, *Aegilops variabilis*; Ca, *Camptotheca acuminata*; Cr, *Catharanthus roseus*; Ms, *Mitragyna speciosa*; Opu, *Ophiorriza pumila*; Os, *Oryza sativa*; Pep, *Capsicum annuum*; Pl, *Peonia lactiflora*; Rv, *Rauvolfia verticillata*; Wc, *Withania coagulans*; Ws, *Withania somnifera*; Sl, *Solanum lycopersicum*.

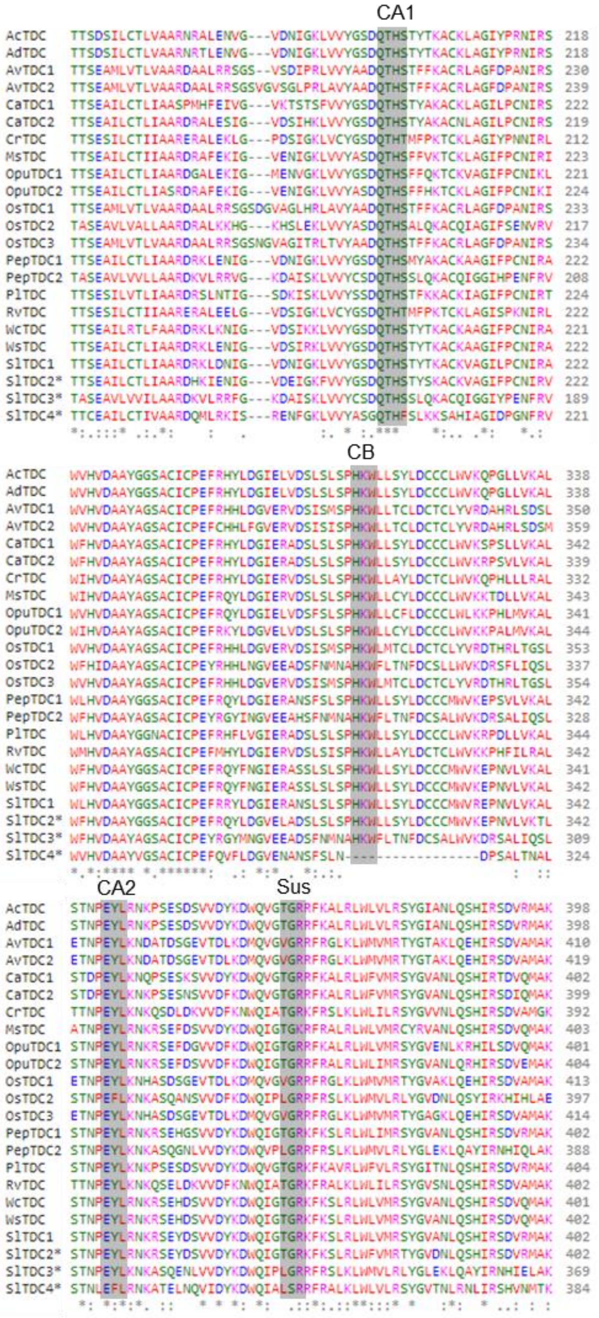

Supplementary Figure 2. Phylogenetic analysis of putative SlTDCs and their relationship to other plant TDCs and TyDCs. The percentage of replicate trees in which the associated taxa clustered together in the bootstrap test (1000 replicates) is shown next to the branches. Color code: green diamonds = functionally characterized TDCs; yellow diamonds = putative TDCs reported in the literature as protein orthologs of characterized plant TDCs; red diamonds = SlTDC candidates; light-blue triangles = functionally characterized plant TyDCs. The proteins in this phylogenetic tree are described in more detail in Supplementary Table 1.

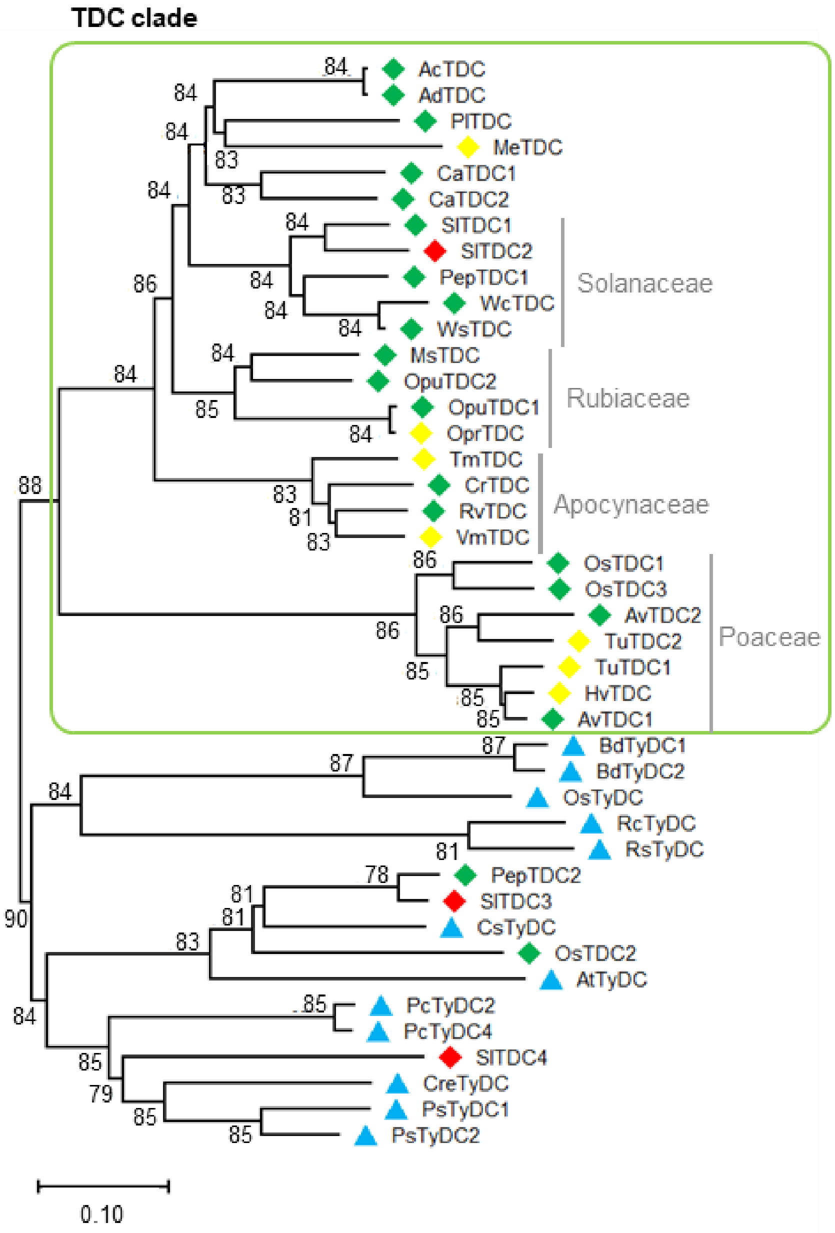

Supplementary Figure 3. Expression levels of *SlTDCs* reported in transcriptomic databases. Transcript levels of *SlTDC1 (Solyc07g054860)*, *SlTDC2* (*Solyc07g054280*), *SlTDC3* (*Solyc09g064430*) and Solyc03044120 (*Solyc03g044120*) in different organs and developmental stages of wild-type tomato cultivars Heinz 1706 (A) and Micro-Tom (B) based on microarray analysis (Tomato Genome Consortium, 2012) and RNA-Seq data (Tom-Express). Legend for B: DPA = days post anthesis; DPG = days post germination; MG = mature-green.

(A)

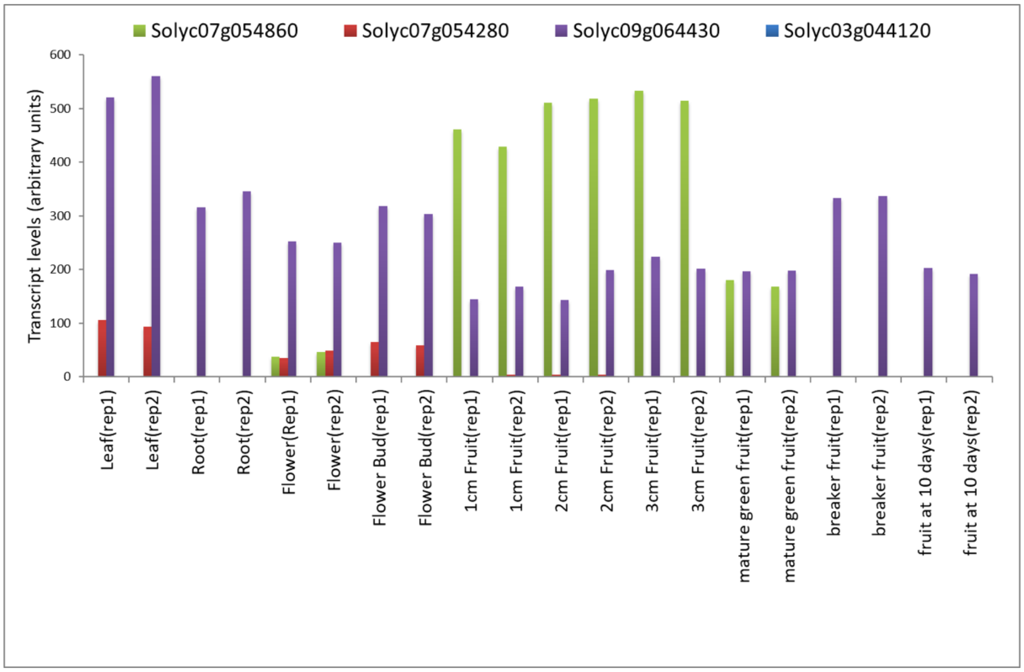

(B)

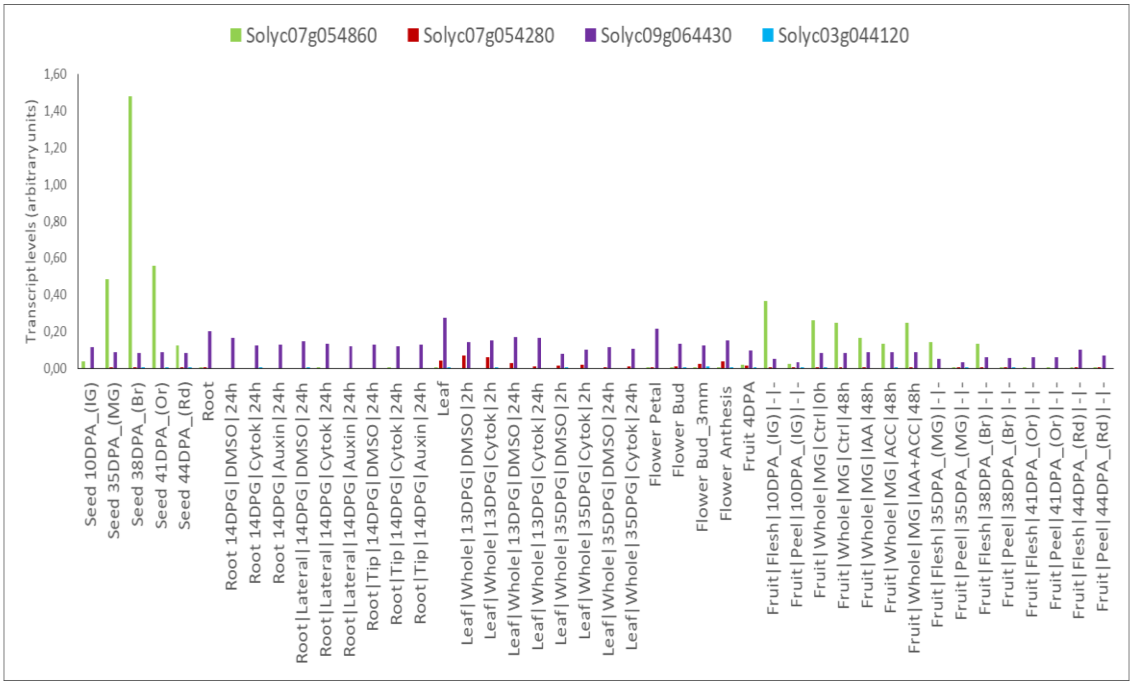

Supplementary Figure 4. Integration to Figure 2. Levels of other SlTDC1/SlTDC2-specific metabolites in the infiltrated leaves. AU = arbitrary units. Data are means ± standard deviations (n = 3).

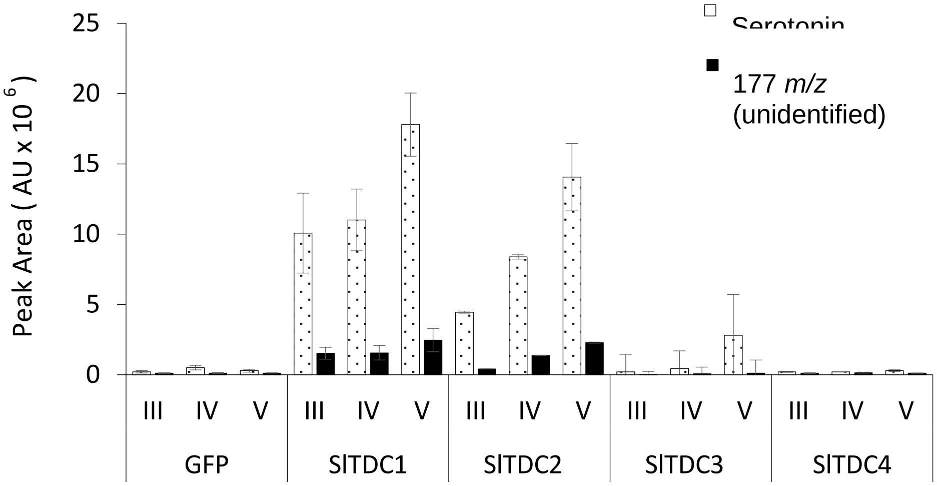

Supplementary Figure 5. Integration to Figure 3. Levels of the unidentified metabolite (222 *m/z*). AU = arbitrary unit. Data are means ± standard deviations (n = 3).

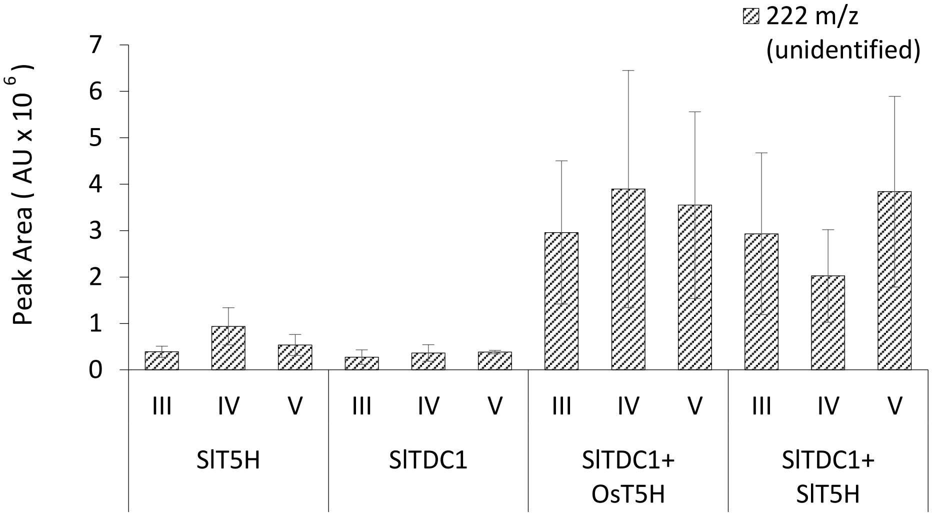

Supplementary Figure 6. Pearson Correlation Analysis of qRT-PCR and metabolomics data. Heat map representation of the Pearson correlation coefficients (PCCs) calculated for genes and metabolites of the tomato serotonin pathway based on our metabolomics and gene expression datasets.

**Roots**	*SlTDC2*	*SlTDC3*	*SlT5H*	TAM
SlTDC3	0.52			
SlT5H	0.71	0.87		
TAM	-0.47	-0.82	-0.68	
SER	0.07	0.79	0.55	-0.78

**Basal leaves**	*SlTDC2*	*SlTDC3*	*SlT5H*	TAM
SlTDC3	0.08			
SlT5H	0.71	0.22		
TAM	0.01	-0.63	-0.26	
SER	0.02	-0.78	-0.43	0.83

**Upper leaves**	*SlTDC2*	*SlTDC3*	*SlT5H*	TAM
SlTDC3	0.53			
SlT5H	0.49	0.81		
TAM	0.17	-0.04	0.44	
SER	0.00	0.21	-0.19	-0.77

**Developing fruit**	*SlTDC1*	*SlTDC2*	*SlTDC3*	*SlT5H*	TAM
SlTDC2	-0.49				
SlTDC3	0.16	-0.24			
SlT5H	0.31	-0.33	0.81		
TAM	0.76	-0.72	0.47	0.54	
SER	-0.18	-0.42	0.60	0.70	0.28

**Ripening fruit**	*SlTDC1*	*SlTDC2*	*SlTDC3*	*SlT5H*	TAM
SlTDC2	0.12				
SlTDC3	-0.27	0.37			
SlT5H	0.94	0.04	-0.21		
TAM	-0.81	0.18	0.67	-0.77	
SER	0.37	0.59	0.21	0.22	0.05

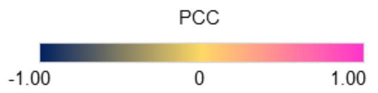

Supplementary Tables

Supplementary Table 1. Characterized and putative plant TDCs and TyDCs used in computational analysis. List of plant TDCs and TyDCs showing the species, the abbreviation used in the paper, the NCBI accession numbers, and the organism used for functional characterization (putative plant TDCs identified in silico are also shown). Asterisks indicates accession numbers from the Kiwifruit Genome Database (†) or the Kazusa Full-length Tomato cDNA Database (‡).

	Organism	Abbreviation	NCBI code	Reference	Expression host
characterized TDCs	*Actinidia chinensis*	AcTDC	Achn173261†	Commisso et al., 2019	*Nicotiana benthamiana*
	Actinidia deliciosa	AdTDC	QBK17431	Commisso et al., 2019	*N. benthamiana*
	Aegilops variabilis	AvTDC1<br>AvTDC2	Not deposited<br>Not deposited	Li et al., 2016	*Escherichia coli*
	Camptotheca acuminata	CacTDC1<br>CacTDC2	AAB39708<br>AAB39709	López-Meyer et al., 1997	*E. coli*
	Capsicum annuum	PepTDC1<br>PepTDC2	NP_001312016<br>ACN62126	Park et al., 2009	*E. coli*
	Catharanthus roseus	CrTDC	AAA33109	De Luca et al., 1989	*E. coli*
	Mytragina speciosa	MsTDC	AEQ01059	Charoonratana et al., 2013	*E. coli*
	Ophiorrhiza pumila	OpuTDC1	BAC41515	Yamazaki et al., 2003	*E. coli*
		OpuTDC2	QLQ34389	You et al., 2020	*E.coli*
	Oryza sativa	OsTDC1<br>OsTDC2	BAG91223<br>BAG95977	Kang et al.,2007a	*E. coli*
		OsTDC3	NP_001060969	Byeon et al.,2014	*O. sativa*
	Peonia lactiflora	PlTDC	ART46241	Zhao et al., 2019	*E.coli*
	Rauvolfia verticillata	RvTDC	ADL28270	Liu et al., 2012	*E. coli*
	Solanum lycopersicum	SlTDC1	LEFL2019I23‡	Tsunoda et al., 2021	*S. lycopersicum*
	Withania coagulans	WcTDC	not deposited	Jadaun et al., 2017	*E. coli*
	Withania somnifera	WsTDC	not deposited	Jadaun et al., 2020	*E. coli*
putative TDCs	*Hordeum vulgare*	HvTDC	BAD11769	Li et al., 2016	
	Manihot esculenta	MeTDC	ANN44945	Wei et al., 2016	
	Ophiorrhiza prostrata	OprTDC	ABU40982	De Masi et al., 2017	
	Tabernaemontana elegans	TeTDC	AEY82396	De Masi et al., 2017	
	Triticum urartu	TuTDC1<br>TuTDC2	EMS51668<br>EMS68447	Li et al., 2016	
	Vinca minor	VmTDC	AEY82397	De Masi et al., 2017	
characterized TyDCs	*Arabidopsis thaliana*	AtTyDC	Q8RY79	Lehmann & Pollmann, 2009	
	Brachypodium distachyon	BdTyDC1<br>BdTyDC2	KQK09947<br>KQK09953	Noda et al., 2015	
	Citrus reshni	CreTyDC	ACX29991	Bartley et al., 2010	
	Oryza sativa	OsTyDC	BAG89694	Park et al., 2012	
	Petroselinum crispum	PcTyDC2<br>PcTyDC4	AAA33860<br>AAA33863	Kawalleck et al., 1993	
	Papaver somniferum	PsTyDC1<br>PsTyDC2	AAA62346<br>AAA62347	Facchini & De Luca, 1995	
	Rhodiola crenulata	RcTyDC	AFN89854	Lan et al., 2013	
	Rhodiola sachalinensi	RsTyDC	ABF06560	Zhang et al., 2011	

Supplementary Table 2. Primers used for the specific amplification of *SlTDC1-4* and *SlT5H* cDNA regions or CDS using KAPA HiFi DNA polymerase (Roche, Basel, Switzerland). CACC bases at the beginning of each forward primer allow directional cloning in the pENTR/D-TOPO vector.

	Forward primer (5′→3′)	Reverse primer (5′→3′)
SlTDC1	CACCATGGGAAGCCTTGATTCCA	TTAAAACACACTTTTTCTCAGCAAAC
SlTDC2	CACCAACTACCAATACTTAGTCTTTCCCCC	AACAATCACAAATGCCTATACATAAAT
SlTDC3	CACCATGGAAGGGGGATTGAAG	TTAACATTTACTTAACAATGTAGCAGC
SlTDC4	CACCGCTAACCATTTATTTTTCTTTCAAGG	TTGATTTAGCAGGGCATTGG
SlT5H	CACCATGGAAGCATCAATTCTACAGCTAC	TTACAACTTGTTGATGGTTGGAAC

Supplementary Table 3. PCR conditions for *SlTDCs* and *SlT5H* cloning from Micro-Tom total cDNA. †The presence of the 5′-CACC-3′ tag in the forward primer adds four extra nucleotides to the cloned sequences.

		SlTDC1	***SlTDC2***	***SlTDC3***
	SGNdb ID	*Solyc07g054860*	*Solyc07g054280*	*Solyc09g064430*
	Source	cDNA	cDNA	cDNA
	Primer design	5'-CDS/3'-CDS	5'-UTR/3'-UTR	5'-UTR/3'-CDS
	Lenght (bp) †	1519	1705	1468
	Initial denaturation	95 °C, 3 min		
x 5	Denaturation	98 °C, 20 s		
	Annealing	60 °C, 45 s	59 °C, 45 s	60 °C, 45 s
	Extension	72 °C, 1 min 32 s	72 °C, 1 min 42 s	72 °C, 1 min 28 s
x 25	Denaturation	98°C, 20 sec		
	Annealing	65 °C, 45 s	61 °C, 45 s	64 °C, 45 s
	Extension	72 °C, 1 min 32 s	72 °C, 1 min 42 s	72 °C, 1 min 28 s
	Final extension	72 °C, 5 min		

		SlTDC4	
	SGNdb ID	*Solyc03g044120*	
	Source	cDNA	genomic DNA
	Primer design	5'-UTR/3'-UTR	5'-UTR/3'-UTR
	Lenght (bp) †	1525	1577
	Initial denaturation	95 °C, 3 min	
x 5	Denaturation	98 °C, 20 s	
	Annealing	54 °C, 45 s	54 °C, 60 s
	Extension	72 °C, 1 min 32 s	72 °C, 1 min 32 s
x 25	Denaturation	98 °C, 20 s	
	Annealing	59 °C, 45 s	59 °C, 60 s
	Extension	72 °C, 1 min 32 s	72 °C, 1 min 32 s
	Final extension	72°C, 5 min	

	SlT5H
SGNdb ID	*Solyc09g014900*
Source	cDNA
Primer design	5'-CDS/3'-CDS

	Lenght (bp) †	1492
	Initial denaturation	95 °C, 3 min
x 30	Denaturation	98 °C, 20 s
	Annealing	56 °C, 45 s
	Extension	72 °C, 1 min 30 s
	Final Extension	72 °C, 5 min

Supplementary Table 4. Primers designed with PRIMER3 (https://primer3.ut.ee/) used for the amplification in qRT-PCR of *SlTDC1-3*, *SlT5H* and *SlCAC* cDNA sequences (amplicon size is reported among brackets near the name of each gene; Tm, melting temperature).

	Forward primer (5′→3′)	Reverse primer (5′→3′)
SlTDC1 *(104 bp)*	CCACTTCCACTACAGCCGTCG (Tm = 62.99 °C)	ACATGCGCTCCCTCCATAAGC (Tm = 62.85 °C)
SlTDC2 *(100 bp)*	TCCGATCCCATGTTTGATGTCGT (Tm = 62.25 °C)	ACTTGGCCCGATGTAGCTCAAAA (Tm = 62.43 °C)
SlTDC3 *(103 bp)*	CCGGGGCTATATGAATGGTGTCG (Tm = 63.03 °C)	AGGCGAGAGACCCAGTTCCTG (Tm = 63.56 °C)
SlT5H *(105 bp)*	CGGCCAAATTCCGACTCTAA (Tm = 62.47 °C)	TGAGCTGCAATGATTTGTGGA (Tm = 62.70 °C)
SlCAC *(121 bp)*	GGGTTGTTACATCACCAAAGC (Tm = 62.48 °C)	GTGCTGGTGTGATTGCATCC (Tm = 62.85 °C)

Supplementary Table 5. Indolamines quantification in Micro-Tom plants. Quantities of tryptamine and serotonin detected in Micro-Tom vegetative organs and flowers, expressed as ng g^{-1} FW. Data are means ± SD (n = 3).

			**Tryptamine (ng g^{-1} FW)**			**Serotonin (ng g^{-1} FW)**		
		Id	Mean		SD	Mean		SD
Roots	Primary root	PR	1.60	±	0.63	183.15	±	88.25
	Proximal secondary root	PSR	3.35	±	0.83	146.57	±	28.54
	Distal secondary root	DSR	8.34	±	1.44	27.19	±	4.44
Stem	Hypocotyl	Hy	23.92	±	4.89	2808.37	±	88.61
	Internode 1	In1	76.33	±	7.10	6365.03	±	501.34
	Internode 2	In2	83.90	±	6.78	3695.51	±	146.27
	Internode 3	In3	76.55	±	5.41	2848.10	±	439.97
	Internode 4	In4	54.49	±	16.26	2184.30	±	359.12
	Internode 5	In5	55.64	±	12.65	2177.71	±	185.88
	Internodes 6+7	In6+In7	63.79	±	9.44	1280.63	±	606.75
	Cotyledons	Co	11.37	±	6.69	1746.21	±	295.30
Leaves	First leaves	FL	73.45	±	2.59	4258.30	±	515.44
	Leaves 1	L1	68.66	±	12.42	5809.25	±	680.17
	Leaves 2	L2	82.37	±	15.49	7169.12	±	487.78
	Leaves 3	L3	127.30	±	25.57	8592.63	±	370.54
	Leaves 4	L4	217.38	±	38.39	9322.02	±	1377.22
	Leaves 5	L5	221.97	±	27.31	4428.07	±	1102.46
	Leaves 6	L6	337.60	±	21.12	1218.88	±	242.74
Flower	Flower buds	FB	95.43	±	18.38	737.16	±	171.25
	Flower (anthesis)	FA	123.07	±	39.03	758.54	±	538.48

Supplementary Table 6. Indolamines quantification in Micro-Tom fruits. Quantities of tryptamine and serotonin detected in Micro-Tom fruits at various developmental and ripening stages, expressed as µg g^{-1} FW and as content (µg) per whole fruit (normalized to berry weight). Data are means ± SD (n = 3).

		Id	Berry weight (g)	**Tryptamine (µg g^{-1} FW)** Mean		SD	**Serotonin (µg g^{-1} FW)** Mean		SD	**Tryptamine (µg per fruit)** Mean		SD	**Serotonin (µg per fruit)** Mean		SD
Fruit	Immature green	**IG**	1.93	1.11	±	0.16	0.18	±	1.93	2.14	±	0.31	0.34	±	0.18
	Mature green	**MG**	3.25	2.00	±	0.50	2.24	±	3.25	6.49	±	1.63	7.29	±	0.52
	Breaker	**B**	3.31	1.53	±	0.11	5.58	±	3.31	5.06	±	0.36	18.46	±	2.96
	Turning	**T**	3.45	2.12	±	0.39	5.17	±	3.45	7.33	±	1.34	17.86	±	1.60
	Ripe	**R**	4.06	1.27	±	0.35	3.37	±	4.06	5.14	±	1.44	13.69	±	3.10
	Over-ripe	**OR**	4.10	0.40	±	0.14	5.30	±	4.10	1.66	±	0.57	21.72	±	1.62

Supplementary Table 7. Indolamines quantification in Micro-Tom dissected fruits. Quantities of tryptamine and serotonin detected in different tissues of Micro-Tom fruits at the unripe (IG+MG) and ripe (R+OR) phases, expressed as µg g^{-1} FW. Data are means ± SD (n = 3).

		Id	**Tryptamine (µg g^{-1} FW)** Mean		SD	**Serotonin (µg g^{-1} FW)** Mean		SD
Unripe fruit (IG+MG)	Exocarp	**Ec**	1.06	±	0.20	4.24	±	0.44
	Mesocarp	**Mc**	4.17	±	0.33	7.81	±	1.44
	Seeds	**S**	7.57	±	0.19	5.51	±	1.09
Ripe fruit (R+OR)	Exocarp	**Ec**	1.37	±	0.15	2.89	±	0.08
	Mesocarp	**Mc**	2.65	±	0.22	4.99	±	0.58
	Seeds	**S**	8.33	±	0.92	9.24	±	1.54

Supplementary Information 1. Retrieval of Micro-Tom *SlTDCs* genes

We used the CDS of the SlTDCs deposited in SGNdb, which are referred to tomato cultivar Heinz 1706, to search the Kazusa Full-length Tomato cDNA database, KafTom (http://www.pgb.kazusa.or.jp/kaftom/blast.html) (Aoki et al., 2010) in order to retrieve orthologs from the cultivar Micro-Tom. Blastn searches revealed that *SlTDC1* and *SlTDC3* matched two full-length cDNA clones (LEFL2019I23 and LEFL2001AC09, respectively) whereas *SlTDC2* was 89.6% identical to clone LEFL2019I23, but contained many mismatches, including sites in the region upstream of the putative CDS (see figure below). The *SlTDC4* CDS did not match any deposited Micro-Tom cDNA sequence. The primers for *SlTDC1* and *SlTDC3* were thus designed to match the CDS ends, whereas the primers for *SlTDC2* and *SlTDC4* were designed to match the 5′ and 3′ untranslated regions (UTRs).

In silico structural analysis of *SlTDC2*: Clustal Omega alignment of the best-matching clone LEFL2019I23 (Kazusa Full-length Tomato cDNA database) with the *SlTDC2* coding sequence.

```
LEFL2019I23      atacttgttcttctgtgatcaatctcattattttcactgtattaacgtaccttaaattgt     60
SlTDC2           ------------------------------------------------------------     0

LEFL2019I23      tccaaaaatcaaatcatgggaagccttgattccaataacagctctccaacccaaaccaac     120
SlTDC2           ---------------ATGGGAACCCTTAATTCAAATAACAACCCTCAAACCCAATCCAAC     45
                                ******* **** **** ******* * *** ******* *****

LEFL2019I23      gttccaaaattcaacccgcttgacccggaagaattccggacccaagcccatcaaatggtg     180
SlTDC2           TTCCCAAAATTCAACCCGCTTGACCCGGAAGAATTCCGTACCCAAGCCCATCAAATGGTG     105
                  * *********************************** *********************

LEFL2019I23      gacttcattgctgattactacaagaatattgagacctacccggttctaagccaagtcgaa     240
SlTDC2           GACTTCATTGCTGATTACTACAAGAATATTGAGTCCTACCCGGTTCTAAGTCAAGTCGAA     165
                 ********************************* **************** *********

LEFL2019I23      ccgggttatctccgtactcaattacccgaaaatgccccctactgccccgaaccattcgag     300
SlTDC2           CCCGGTTATCTTCGTACCCAATTACCCGAAAACGCCCCTAATCGACCCGAATCATTCGAT     225
                 ** ******** ***** ************** *****  *  * ****** *******

LEFL2019I23      gcaattatgaaagatgtccacaaccatattgtccccggtatgacccattggttgagcccg     360
SlTDC2           TTAATTATGAAAGATGTCCAAAACCATATTATCCCGGGTATGACCCATTGGCTAAGCCCG     285
                   ****************** ********* **** *************** * ******

LEFL2019I23      aatttcttcgcattttttccagctactgttagttccgcagcgttcattggtgaaatgctt     420
SlTDC2           AATTTCTTCGCATTTTTTCCAGCTACTGTTAGCTCCGCTGCGTTTCTAGGTGAAATGCTT     345
                 ******************************** ***** *****  * ************

LEFL2019I23      tgcaattgtttcaactccgtcgggtttaactggctggcttcgccggccatgacggagttg     480
SlTDC2           TGCAATTGTTTCAACTCCGTCGGATTTAATTGGCTGGCTTCGCCAGCCATGACGGAGTTG     405
                 *********************** ***** ************** ***************

LEFL2019I23      gaaatgatagtcatggactggctcgctaatatgttgaaattaccaaaagccttcatgttt     540
SlTDC2           GAAATGGTAGTCATGGACTGGCTTGCTAATACGTTGAAATTACCAAAAACTTTCATGTTT     465
                 ****** **************** ******* **************** * *********

LEFL2019I23      tctggcacgggtggtggtgtacttcaaagtacaaccagtgaagcgatcctatgcacgtta     600
SlTDC2           TCTGGCACGGGTGGTGGTGTACTACAAAGTACAACTAGTGAAGCTATACTGTGTACGTTA     525
                 *********************** *********** ******** ** ** ** ******

LEFL2019I23      attgctgcacgtgatcgtaaactcgataacataggcgttgataacatcggaaagcttgta     660
SlTDC2           ATCGCTGCGCGTGATCATAAGATCGAGAATATAGGTGTTGATGAGATAGGAAAATTTGTA     585
                 ** ***** ******* ***  **** ** ***** ****** * ** *****  *****

LEFL2019I23      gtctatggttctgancaaacgcattctacgtataccaaagcctgnnnggtagctggtatt     720
SlTDC2           GTCTACGGTTCTGATCAAACTCACTCTACTTATAGCAAAGCCTGCAAGGTAGCTGGTATT     645
                 ***** ******** ***** ** ***** **** *********   *************

LEFL2019I23      ttaccatgcaatattcgtgngg--------------------------------------     742
SlTDC2           TTCCCATGCAATATTCGTGTGGTACCAACTTGTATTGAAAGCGATTTCGCTTTATCTCCT     705
                 ** **************** **
```

Supplementary Information 2. *In silico* structural analysis of *SlTDC4* clone

The alignment of the cloned *SlTDC4* Micro-Tom genomic DNA sequence and the corresponding *Solyc03g044120* sequence in SGNdb (on the left in the figure below) revealed six nucleotide substitutions, and thus 99.58% identity. Translation *in silico* (on the right in the figure below) suggested that the substitutions potentially resulted in five amino acid changes compared to the Solyc03g044120 protein but none of the variant sites affected residues responsible for TDC activity.

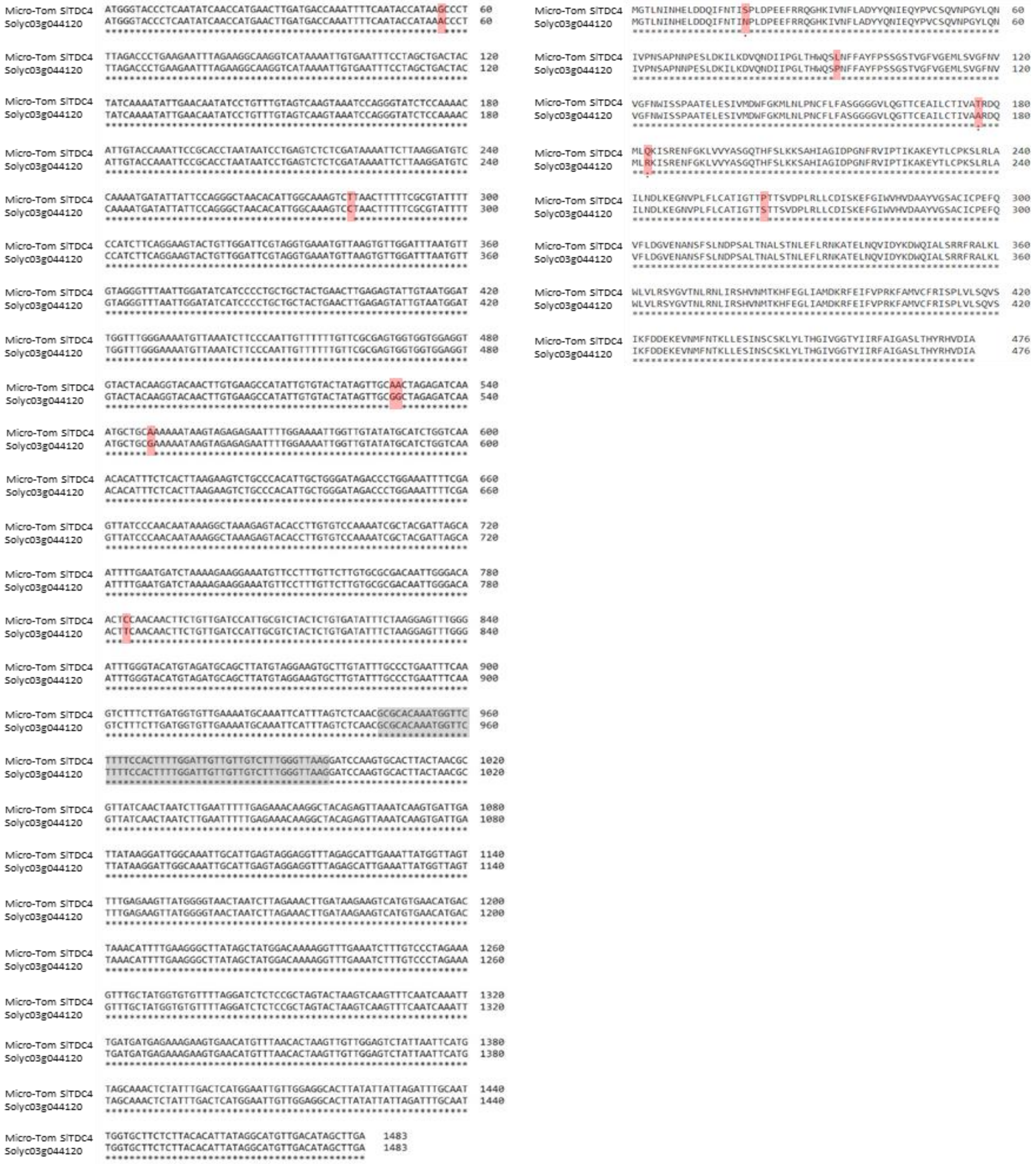

Supplementary Information 3. Further characterization of *SlTDC4*

The absence of tryptamine in plants infiltrated with *SlTDC4* could reflect the failure of gene expression or the inactivity of the protein, or the protein may function as a TyDC instead of a TDC. To rule out the first explanation, the strong expression of *SlTDC4* in the infiltrated plants was confirmed by RT-PCR. Sequencing the Micro-Tom SlTDC4 clone revealed the persistence of a 52 bp intron sequence including a potential cryptic TAA stop codon, suggesting that the lack of activity may reflect premature protein truncation followed by incomplete or incorrect folding The figure below shows the Clustal Omega alignment of (A) the nucleotide sequences of the *SlTDC4* cDNA clone (RT-PCR of the leaves of *N. benthamiana* plants infiltrated with pK7WG2.*SlTDC4*) and the *Solyc03g044120* coding and genomic sequences, and (B) the *in silico* translated SlTDC4 clone (early truncated protein) and the Solyc03g044120 protein. Yellow boxes in (A) and (B) highlight, respectively, an intron and the corresponding polypeptide, and the red box indicates a stop codon.

(A)

```
SlTDC4-cDNA           ATTTGGGTACATGTAGATGCAGCTTATGTAGGAAGTGCTTGTATTTGCCCTGAATTTCAA   900
Solyc03g044120-CDS    ATTTGGGTACATGTAGATGCAGCTTATGTAGGAAGTGCTTGTATTTGCCCTGAATTTCAA   900
Solyc03g044120-gDNA   ATTTGGGTACATGTAGATGCAGCTTATGTAGGAAGTGCTTGTATTTGCCCTGAATTTCAA   900
                      ************************************************************

SlTDC4-cDNA           GTCTTTCTTGATGGTGTTGAAAATGCAAATTCATTTAGTCTCAACGCGCACAAATGGTTC   960
Solyc03g044120-CDS    GTCTTTCTTGATGGTGTTGAAAATGCAAATTCATTTAGTCTCAA----------------   944
Solyc03g044120-gDNA   GTCTTTCTTGATGGTGTTGAAAATGCAAATTCATTTAGTCTCAACGCGCACAAATGGTTC   960
                      ********************************************

SlTDC4-cDNA           TTTTCCACTTTTGGATTGTTGTTGTCTTTGGGTTAAGGATCCAAGTGCACTTACTAACGC   1020
Solyc03g044120-CDS    ------------------------------------CGATCCAAGTGCACTTACTAACGC   968
Solyc03g044120-gDNA   TTTTCCACTTTTGGATTGTTGTTGTCTTTGGGTTAAGGATCCAAGTGCACTTACTAACGC   1020
                                                           ***********************
```

(B)

```
SlTDC4           MGTLNINHELDDQIFNTISPLDPEEFRRQGHKIVNFLADYYQNIEQYPVCSQVNPGYLQN   60
Solyc03g044120   MGTLNINHELDDQIFNTINPLDPEEFRRQGHKIVNFLADYYQNIEQYPVCSQVNPGYLQN   60
                 ******************.*****************************************

SlTDC4           IVPNSAPNNPESLDKILKDVQNDIIPGLTHWQSLNFFAYFPSSGSTVGFVGEMLSVGFNV   120
Solyc03g044120   IVPNSAPNNPESLDKILKDVQNDIIPGLTHWQSPNFFAYFPSSGSTVGFVGEMLSVGFNV   120
                 ********************************* **************************

SlTDC4           VGFNWISSPAATELESIVMDWFGKMLNLPNCFLFASGGGGVLQGTTCEAILCTIVATRDQ   180
Solyc03g044120   VGFNWISSPAATELESIVMDWFGKMLNLPNCFLFASGGGGVLQGTTCEAILCTIVAARDQ   180
                 ********************************************************:***

SlTDC4           MLQKISRENFGKLVVYASGQTHFSLKKSAHIAGIDPGNFRVIPTIKAKEYTLCPKSLRLA   240
Solyc03g044120   MLRKISRENFGKLVVYASGQTHFSLKKSAHIAGIDPGNFRVIPTIKAKEYTLCPKSLRLA   240
                 **:*********************************************************

SlTDC4           ILNDLKEGNVPLFLCATIGTTPTTSVDPLRLLCDISKEFGIWVHVDAAYVGSACICPEFQ   300
Solyc03g044120   ILNDLKEGNVPLFLCATIGTTSTTSVDPLRLLCDISKEFGIWVHVDAAYVGSACICPEFQ   300
                 ********************* **************************************

SlTDC4           VFLDGVENANSFSLNAHKWFFSTFGLLLSLG-----------------------------   331
Solyc03g044120   VFLDGVENANSFSLNDPSALTNALSTNLEFLRNKATELNQVIDYKDWQIALSRRFRALKL   360
                 ***************  . : .::.  *.:

SlTDC4           ------------------------------------------------------------   331
Solyc03g044120   WLVLRSYGVTNLRNLIRSHVNMTKHFEGLIAMDKRFEIFVPRKFAMVCFRISPLVLSQVS   420

SlTDC4           --------------------------------------------------------   331
Solyc03g044120   IKFDDEKEVNMFNTKLLESINSCSKLYLTHGIVGGTYIIRFAIGASLTHYRHVDIA   476
```

Therefore, we tested the activity of the original SGNdb sequence (Heinz 1706 *SlTDC4*; *Solyc03g044120*) by cloning the CDS in binary vector pK7WG2 and repeating the transient expression experiments. However, no tryptamine was detected despite the verification of *Solyc03g044120* expression by RT-PCR (figure below), suggesting that SlTDC4 does not function as a TDC.

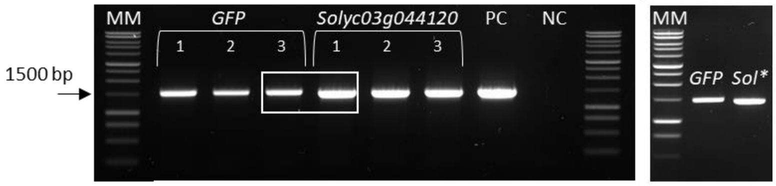

RT-PCR of *Solyc03g044120*. Gel electrophoresis of cDNA amplified from infiltrated *Nicotiana benthamiana* leaves. PC = positive control (pK7WG2.*Solyc03g044120* plasmid), NC = negative control, MM = 1-kb molecular ladder. Right hand panel shows a magnification of the white box (**Solyc03g044120*).

This combination of computational and experimental evidence confirmed that neither Micro-Tom SlTDC4 nor the Heinz 1706 Solyc03g044120 sequence are functional TDCs, although they were annotated as TDC candidates (Pang et al., 2018). These sequences may be pseudogenes because they are not expressed in tomato under any conditions, they lack the lysine residue needed for cofactor binding (compromising their putative decarboxylase activity), and the 52-bp intronic region of the Micro-Tom SlTDC4 gene was not spliced out once transcribed in *N. benthamiana* suggesting the loss of functional splice sites during evolution, which is a typical feature of some classes of pseudogenes (Zhu and Niu, 2013).

Curiously, RT-PCR analysis revealed the amplification of a ~1500-bp product in the GFP-expressing control plants, suggesting that *N. benthamiana* expresses a gene homologous to *Solyc03g044120*. Direct sequencing of the gel-purified amplicon using the *SlTDC4* forward primer yielded a partial sequence (885 bp) that was used as a blastn search query against the SGNdb and the *Nicotiana benthamiana* genome website (https://www.nbenth.com/). The former returned a 1431-bp CDS (*Niben101Scf02857g02001.1*) annotated as a putative TyDC, whereas the latter returned a 1867-bp cDNA including the CDS as well as 5′and 3′ UTRs (Nbv6.1trP5228). The two sequences were translated in silico and alignment revealed 100% identity, indicating they correspond to a unique protein. ClustalO alignment between this sequence and Solyc03g044120 revealed 75.6% identity (Figure C), suggesting that an ortholog of Solyc03g044120 is expressed *N. benthamiana*.

```
Solyc03g044120              MGTLNINHELDDQIFNTINPLDPEEFRRQGHKIVNFLADYYQNIEQYPVCSQVNPGYLQN     60
Niben101Scf02857g02001      -------------------------------MIVNFLADYYENIEKYPVCSQVNPGYLQK     29
                                                            *********:***:*************:

Solyc03g044120              IVPNSAPNNPESLDKILKDVQNDIIPGLTHWQSPNFFAYFPSSGSTVGFVGEMLSVGFNV     120
Niben101Scf02857g02001      LVPNSAPDNPEPLEKILHDVKRDIIPGITHWQSPNFFAYFPSSGSTAGFLGEMLSVGFNV     89
                            :******:*** *:***:**:.*****:******************.**:**********

Solyc03g044120              VGFNWISSPAATELESIVMDWFGKMLNLPNCFLFASGGGGVLQGTTCEAILCTIVAARDQ     180
Niben101Scf02857g02001      VGFNWISSPAATELESIVMDWFGKMLNLPNSFLFSGGGGGVLQGTTCEAMLCTIVAARDQ     149
                            ******************************.***:.*************:**********

Solyc03g044120              MLRKISRENFGKLVVYASGQTHFSLKKSAHIAGIDPGNFRVIPTIKAKEYTLCPKSLRLA     240
Niben101Scf02857g02001      MLRKIGRENFDKLVVYISDQTHFSLKKAAHIAGIDPENFRVIPTTKANEYVLCPKSLRLA     209
                            *****.****.***** *.********:******** ******* **:**.*********

Solyc03g044120              ILNDLKEGNVPLFLCATIGTTSTTSVDPLRLLCDISKEFGIWVHVDAAYVGSACICPEFQ     300
Niben101Scf02857g02001      ILKDTKEGMIPLFLCATIGTTSTTSIDPLRSLCEIAKEYGIWVHVDAAYAGSACICPEFQ     269
                            **:* *** :***************:**** **:*:**:**********.**********

Solyc03g044120              VFLDGVENANSFSLN-----------------DPSALTNALSTNLEFLRNKATELNQVID     343
Niben101Scf02857g02001      HFLDGIENANSFSLNAHKWFFSTLDCCCLWVKDPSALTKALSTNPECLRNKATELNQVID     329
                             ****:*********                 ******:***** * *************

Solyc03g044120              YKDWQIALSRRFRALKLWLVLRSYGVTNLRNLIRSHVNMTKHFEGLIAMDKRFEIFVPRK     403
Niben101Scf02857g02001      YKDWQISLSRRFRALKLWLVLRNYGVANLRNLIRSHVNMAKHFEGLIAMDKRFEIFVPRK     389
                            ******:***************.***:************:********************

Solyc03g044120              FAMVCFRISPLVLSQVSIKFDDEKEVNMFNTKLLESINSCSKLYLTHGIVGGTYIIRFAI     463
Niben101Scf02857g02001      FAMVCFRISPLVISRVSTTFDEE-QVNKFNTKLVESINSSGKLYLTHGVVGGIYIIRFAI     448
                            ************:*:** .**:* :** *****:*****..*******:*** *******

Solyc03g044120              GASLTHYRHVDIA---------------     476
Niben101Scf02857g02001      GASLTDYRHVDMAWKVIQDHATRLFLII     476
                            *****.*****:*
```

In silico structural analysis of Solyc03g044120 orthologs. Clustal Omega alignment of the amino acid sequences of Solyc03g044120 (SlTDC4 of cv. Heinz1706) and its putative *N. benthamiana* ortholog (Niben101Scf02857g02001.1).

References:

Aoki, K., Yano, K., Suzuki, A., Kawamura, S., Sakurai, N., Suda, K., Kurabayashi, A., Suzuki, T., Tsugane, T., and Watanabe, M. (2010). Large-scale analysis of full-length cDNAs from the tomato (*Solanum lycopersicum*) cultivar Micro-Tom, a reference system for the Solanaceae genomics. *BMC genomics* **11,** 1-16.

Pang, X., Wei, Y., Cheng, Y., Pan, L., Ye, Q., Wang, R., Ruan, M., Zhou, G., Yao, Z., and Li, Z. (2018). The tryptophan decarboxylase in *Solanum lycopersicum*. *Molecules* **23,** 998.

Zhu, T., and Niu, D.-K. (2013). Frequency of intron loss correlates with processed pseudogene abundance: a novel strategy to test the reverse transcriptase model of intron loss. *BMC biology* **11,** 1-12.
